# MicroRNA-124 and -137 cooperativity controls caspase-3 activity through BCL2L13 in hippocampal neural stem cells

**DOI:** 10.1038/srep12448

**Published:** 2015-07-24

**Authors:** Marijn Schouten, Silvina A. Fratantoni, Chantal J. Hubens, Sander R. Piersma, Thang V. Pham, Pascal Bielefeld, Rob A. Voskuyl, Paul J. Lucassen, Connie R. Jimenez, Carlos P. Fitzsimons

**Affiliations:** 1 Center for Neuroscience, Swammerdam Institute for Life Sciences, University of Amsterdam, SciencePark 904, 1098XH, Amsterdam, The Netherlands; 2 Oncoproteomics Laboratory, Cancer Center, Free University Amsterdam, De Boelelaan 1117, 1081HV, Amsterdam, The Netherlands; 3 Division of Pharmacology, LACDR, Leiden University, Einsteinweg 55, 2333 CC Leiden, The Netherlands; 4 Foundation of Epilepsy Institutes of The Netherlands (SEIN), Achterweg 5, 2103 SW, Heemstede, The Netherlands.

## Abstract

Adult neurogenesis continuously contributes new neurons to hippocampal circuits and the programmed death of a subset of immature cells provides a primary mechanism controlling this contribution. Epileptic seizures induce strong structural changes in the hippocampus, including the induction of adult neurogenesis, changes in gene expression and mitochondrial dysfunction, which may all contribute to epileptogenesis. However, a possible interplay between this factors remains largely unexplored. Here, we investigated gene expression changes in the hippocampal dentate gyrus shortly after prolonged seizures induced by kainic acid, focusing on mitochondrial functions. Using comparative proteomics, we identified networks of proteins differentially expressed shortly after seizure induction, including members of the BCL2 family and other mitochondrial proteins. Within these networks, we report for the first time that the atypical BCL2 protein BCL2L13 controls caspase-3 activity and cytochrome C release in neural stem/progenitor cells. Furthermore, we identify BCL2L13 as a novel target of the cooperative action of microRNA-124 and microRNA-137, both upregulated shortly after seizure induction. This cooperative microRNA-mediated fine-tuning of BCL2L13 expression controls casp3 activity, favoring non-apoptotic caspase-3 functions in NSPC exposed to KA and thereby may contribute to the early neurogenic response to epileptic seizures in the dentate gyrus.

New neurons in the adult dentate gyrus (DG) originate from neural stem/progenitor cells (NSPC) located in the subgranular zone (SGZ) of the DG^1^. The newly generated cells undergo proliferation, selection, migration and neuronal differentiation before they are functionally integrated into hippocampal networks where they contribute to hippocampal functions^2^. In most cases, these stages engage specific cell types in the DG^3^. Under normal conditions, newborn cells are selected by apoptosis shortly after their birth and are rapidly phagocytosed by microglia^4^. Therefore, apoptosis provides a primary mechanisms to control neuronal cell numbers and neuronal circuit formation in the DG^5^,^6^. Adult neurogenesis in the DG is under tight molecular control by cell intrinsic factors, such as specific small non-coding RNAs termed microRNAs^7^ (miRs) which regulate gene expression posttranscriptionaly by recognizing specific mRNAs and targeting them for translational repression and/or cleavage^8^,^9^. Adult neurogenesis is also influenced by environmental factors, such as physical activity, environmental enrichment, and kainic acid–induced seizures^10^.

Adult generated granule neurons may play a substantial role in the development of epilepsy, although their specific contribution remains unclear^11^,^12^. Seizures increase NSPC proliferation in post-seizure animal models of epilepsy including kainic acid (KA)-induced status epilepticus (SE), and in this model suppression of adult neurogenesis increases seizure severity^11^,^13^. A restricted population of neurons born after SE, determined among other factors by the initial SE intensity and resulting activation of caspase3 (casp3) mediated mitochondrial pathways of apoptosis, outlive SE and may contribute to network reorganization and rewiring of hippocampal circuits associated with epileptogenesis^14^,^15^. Recent evidence suggests that caspases play a broader role in NSPC than originally anticipated. Active caspases, particularly casp3, are expressed in different apoptotic and non-apoptotic cells of the forebrain and may play additional roles besides programmed cell death^16^ and contribute to NSPC differentiation^17^. Importantly, NSPC fate appears to be influenced by a balance between anti- and pro-apoptotic B-cell lymphoma 2 (BCL-2) mitochondrial proteins whose expression levels are dictated by several regulatory mechanisms^18^.

Changes in miR expression in epilepsy animal models as well as in the hippocampus of epileptic patients have been identified^19^,^20^,^21^. Interestingly, changes in miR expression may principally impact on proteins involved in neuronal structure, gliosis and apoptosis^22^. Gene regulation by miRs involves a complex interplay between regulatory mechanisms that complicates the elucidation of the actual impact of individual miRNAs^9^. Thus, understanding the coordinated regulation of specific targets by multiple miRs, or miR cooperativity, is key in elucidating the complexity of gene regulation by miRs^23^. Importantly, cooperative miR function could render targets more sensitive to small changes in multiple miRs^24^.

Here, we investigated changes induced in the DG shortly after KA-induced SE (KA-SE), focusing on mitochondrial apoptotic functions in NSPC. Using proteomic, transcriptomic and miR-profiling techniques, we show that particular BCL-2 proteins are downregulated whereas, simultaneously, specific miRs are upregulated. Narrowing down these observations using postnatal hippocampal NSPC cultures as a model to study cell intrinsic molecular mechanisms induced by exposure to KA, we identify the BCL-2 family member BCL-2-Like 13 (BCL2L13) as a novel target of miR-124 and miR-137. We demonstrate that BCL2L13 controls CytC release and casp3 activity in NSPC, and that BCL2L13 expression is regulated by the cooperative action of miR-124 and miR-137.

## Results

### Changes in proteome in the DG after KA-SE

**Figure 1:**
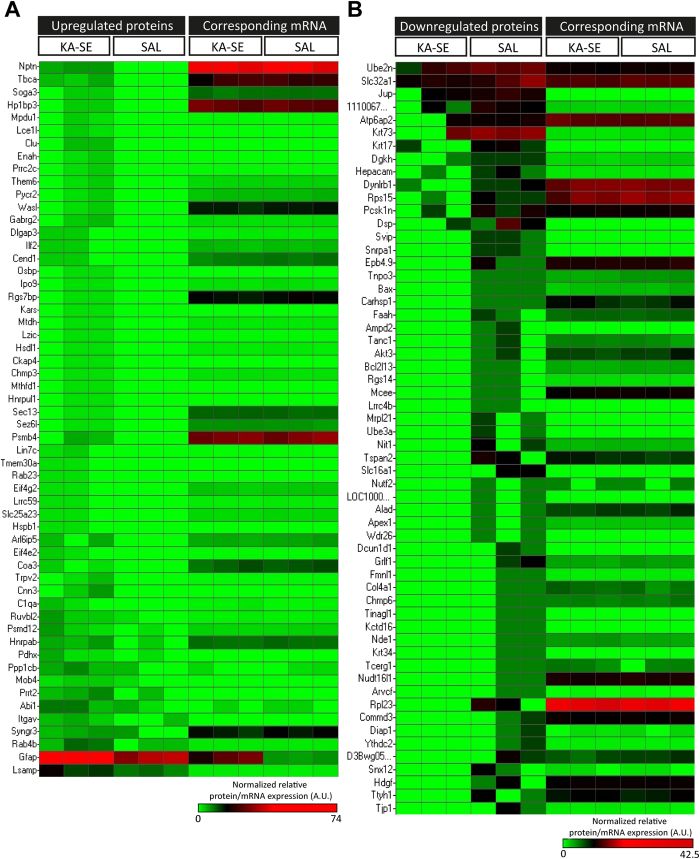
Characteristic proteomic and mRNA expression profile observed in the DG after KA-SE. (**A**) Normalized relative expression of 55 significantly upregulated proteins and corresponding mRNAs in the DG of mice exposed to KA-SE. (**B**) Normalized relative expression of 58 significantly downregulated proteins and corresponding mRNAs in the DG of mice exposed to KA-SE. Up- and Down-regulated proteins were sorted on fold change. Colors represent normalized relative protein/mRNA expression arbitrary units (A.U.), green (<1), black (1) and red (>1).

We detected a significant increase in DCX + cells in the DG of KA-treated animals, starting at 3 days and lasting for at least 7 days after KA-SE, as described before^13^. This neurogenic increase evidenced in DCX + cells was preceded by an increase in the immunoreactivity for glial fibrillary acidic protein (GFAP), which started 1 day after KA-SE and lasted for at least 7 days (Supplementary Fig. 1), likely reflecting reactive astrogliosis reported by others^25^. To understand the relationships between molecular and cellular changes taking place in the DG shortly after SE, we focused on the 3 days after SE time point, when strong changes in gene expression take place^26^. Comparative proteomics between saline (SAL)- and KA-treated animals identified a total of 2327 proteins in the DG, with good sample-to-sample reproducibility in both SAL and KA groups (Supplementary Fig. 1, Supplementary Tables 1 and 2). Beta-binomial analysis identified 114 differentially regulated proteins, with 56 up- and 58 down-regulated in the KA group (Fig. 1 and Supplementary Table 2).

**Figure 2:**
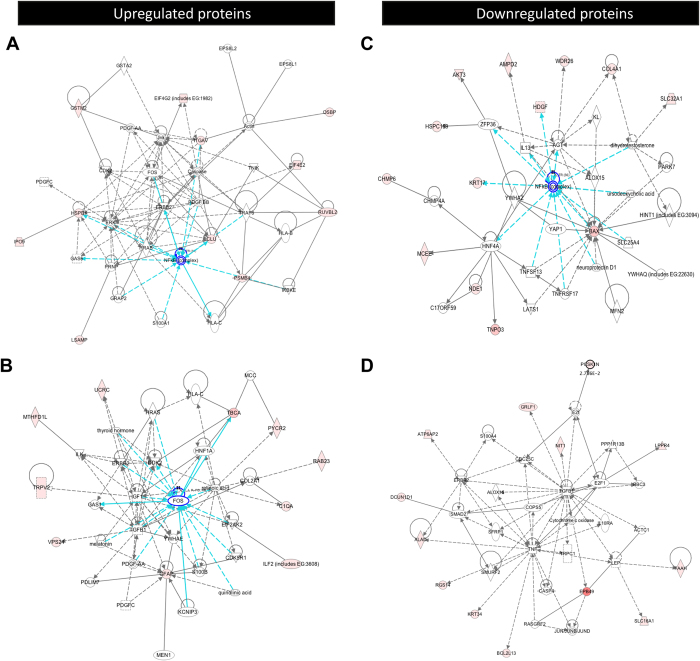
Significantly overrepresented networks containing dysregulated proteins in the DG after KA-SE. (**A**) SON depicting nodes around NFkB, including CLU and other upregulated proteins. (**B**) SON depicting nodes around FOS, including GFAP and other significantly upregulated proteins. (**C**) SON depicting nodes around NFkB, including BAX and other significantly downregulated proteins. (**D**) SON depicting nodes around TNF including BCL2L13 and other significantly downregulated. Gene products (nodes) are represented as standard IPA polygons and relationships with lines (edges) between nodes. Full lines indicate a direct interaction and dashed lines an indirect interaction. Intensity of the node color indicates the degree of regulation (SAL vs. KA) and relationship strength is inversely related to line length. Genes represented by uncolored nodes were not differentially expressed in our experiments and were integrated by the IPA knowledge database. Arrows represent activation while non-arrowed lines binding only.

Global molecular protein networks were identified and visualized using Ingenuity Pathway analysis (IPA, Ingenuity® Systems; Supplementary Fig. 2A,D). These complex networks were reduced into smaller ones, using IPA’s focused gene function (Fig. 2 and Supplementary Fig. 2B–F), revealing one significantly overrepresented network (SON) containing the upregulated protein CLU (Fig. 2A). CLU is upregulated in reactive astrocytes and linked to cell survival^27^, involved in the regulation of postnatal neurogenesis^28^ and executes anti-apoptotic functions by interacting with BAX, blocking CytC release from mitochondria and caspase activation^29^. A second SON included the upregulated protein GFAP (Fig. 2B), linked to astrogliosis. Two others SONs containing upregulated proteins were identified around Nuclear Factor Kappa-B (NFkB) and TNF (Supplementary Figure 2B,C, respectively).

In line with an inhibition of mitochondrial apoptosis pathways suggested by CLU upregulation, two of the SONs containing downregulated proteins contained the proapoptotic BAX and NADH Dehydrogenase Ubiquinone 1 Beta 6 and 7 (NDUFB6, NDUFB7, Fig. 2C and Supplementary Fig. 3F, respectively). BAX is linked to the regulation of adult hippocampal NSPC apoptosis^30^ and NDUFB6 and NDUFB7 are two subunits of the NADH:ubiquinone oxidoreductase complex, involved in ATP generation by oxidative phosphorylation^31^. Interestingly, within the downregulated proteins we identified a third SON containing the BCL-2 protein BCL2L13, (Fig. 2D; Supplementary Table 2). BCL2L13 is a novel atypical BCL-2 protein, localized to mitochondria and which biological function is associated with CytC release and casp3 activation^32^,^33^. A fourth SON containing downregulated proteins was identified around NFkB (Supplementary Fig. 2E). KA-SE-induced downregulation of BAX and BCL2L13 at the protein level was confirmed by western blot (Supplementary Fig. 3A,B).

Next, we used GeneCodis Gene Ontology (GO) analysis to classify the significantly dysregulated proteins into biological processes (BP). Within the BPs overrepresented among upregulated proteins (Supplementary Fig. 4A and Supplementary Table 3), we found transport (containing GABRG2, OSBP, SEC13, SLC25A23, LIN7C, RAB4B and TRPV2), translation (EIF4E2, EIF4G2 and KARS) negative regulation of apoptotis (CLU, ITGAV, MTDH and HSPB1) and nervous system development (ENAH, NPTN and RAB23). GO analysis of the downregulated proteins (Supplementary Fig. 4B and Supplementary Table 4) resulted in the significantly overrepresented BPs transport (SNX12, TNPO3, SLC16A1, CHMP6, SLC32A1, TTYH1 and DYNLRB1), translation (RPL23, RPS15 and MRPL21), nervous system development (JUP, BAX and NDE1), and regulation of apoptotic process (BCL2L13 and BAX). The overrepresentation of BPs linked to apoptosis identified by GO analysis was consistent with BPs identified by IPA, including the downregulated proteins AKT3, BCL2L13 and BAX into the BP mitochondrial apoptosis (Supplementary Fig. 4C). These results suggest that some proteins up- and down-regulated in the DG shortly after KA-SE may converge on the regulation of mitochondrial apoptotic pathways, hallmarked by CytC release from mitochondria and caspase activation.

### Correlation between proteome and transcriptome after KA-SE

We hypothesized that changes in protein levels could be explained by changes in corresponding mRNAs. Gene expression profiling identified a total of 52 genes significantly regulated at the mRNA level with 24 up- and 28 downregulated genes (Supplementary Table 5). Next, Pearson’s correlation analysis was used to analyze protein and mRNA levels^34^. We included in this analysis the 114 differentially expressed proteins in the DG and their corresponding mRNAs (Fig. 1 and Supplementary Tables 2 and 5). Overall, protein levels did not correlate significantly with corresponding mRNA (Pearson r −0.165, p = 0.285; Pearson r −0,215, p = 0.172 for up- and downregulated proteins respectively, Supplementary Fig. 3C,D), suggesting the involvement of posttranscriptional regulatory mechanisms. BAX and BCL2L13 mRNA levels were validated by real time quantitative PCR (RT-QPCR; Supplementary Fig. 3E,F). Unlike BAX protein levels, which corresponded well with its mRNA levels, BCL2L13 protein levels did not (Supplementary Fig. 3C–F).

### Changes in miR expression in the DG after KA-SE

**Figure 3:**
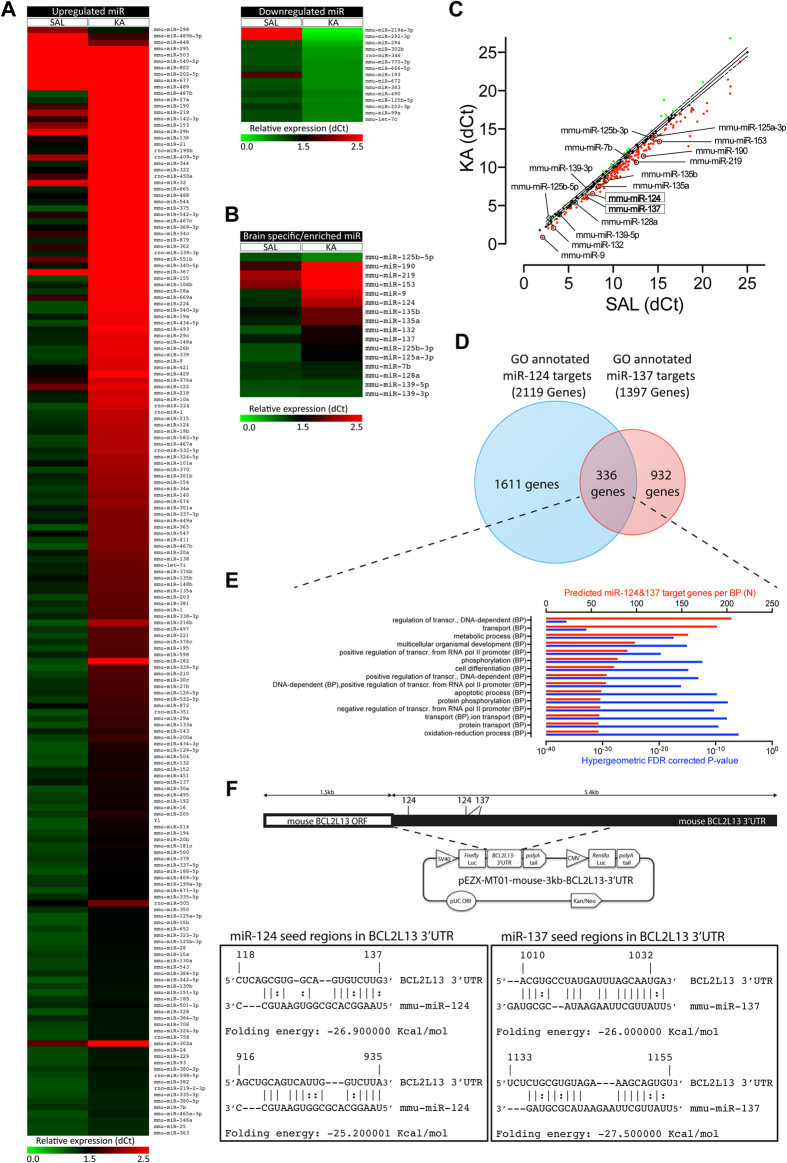
KA-SE induced changes miR expression profile in the DG. (**A**) Relative expression level (dCt) of 189 miRs significantly dysregulated in the DG of mice exposed to KA-SE, (FDR corrected *p* < 0.05; cutoff fold change ≥1.5). (**B**) Relative expression levels (dCt) of a group of brain- enriched or -specific miRs (FDR corrected *p* < 0.05; cutoff fold change ≥1.5). (**C**) Relative expression levels (dCt) of SAL vs. KA showing individual deviations from the RNU6B normalized mean (continuous line) ± SEM (dashed line). Red dots: up- and green dots: down-regulated miRs. Brain- enriched or -specific miR are indicated. (**D**) miR-mRNA interaction prediction mining for miR-124 and miR-137 with ≥3 algorithms positively predicting miR-mRNA binding. (**E**) GO analysis of 336 predicted common targets between miR-124 and miR-137. 61 were annotated in apoptotic processes (Hypergeometric FDR corrected *p* = 1.56*10-10). Red bars: number of annotated members per BP. Blue bars: hypergeometric FDR corrected *p* -values. (**F**) BCL2L13 3′UTR with miR-124 and miR-137 seed regions highlighted. Within BCL2L13 mRNA sequence (NM_153516), the 3′UTR starts in position 1483, after a UAA stop codon, where our 3′UTR position numbering starts. The predicted binding regions at position 916 (miR-124) and 1010 (miR-137) met inclusion criteria for cooperative action described in the text. The reporter plasmid with a truncated BCL2L13 3′UTR (first 3 kb of NM_153516) containing predicted miR-124&137 binding regions is schematically depicted. Calculated folding energy and seed pairing details for miR-124 and miR-137 at the BCL2L13 3′UTR are shown in the boxes.

Next, we explored the possible scenario that some of the discrepancies in protein and mRNA expression could be explained by posttranscriptional regulation by miRs. We detected 277 individual miRs in SAL and KA groups. 189 were differentially expressed, with 173 upregulated and 16 downregulated miRs (Fig. 3A,C; Supplementary Table 6), showing a distinct miR expression profile in the DG after KA-SE. From a group of previously identified brain- enriched or -specific miRs^35^, 16 miRs were detected, with 11 up- and 1 down- regulated (Fig. 3B,C and Supplementary Table 6). Although BCL2L13 is not expressed exclusively in brain tissue^32^, we reasoned that its expression in the brain would likely be regulated by brain-specific miRs. However, this approach may have excluded non-brain specific miRs that may have been more strongly upregulated and thus, could be more potent silencers of BCL2L13. We found multiple predicted binding regions for eight of the 11 upregulated brain specific or enriched miRs, including 2 for miR-124, in the mouse BCL2L13′s 3′UTR (Fig. 3F, Supplementary Fig. 5, Supplementary Tables 10–16).

The prediction of BCL2L13 as common target between miR-124 and 7 other brain- enriched or -specific miRs suggested a coordinated biological action. Therefore, we looked for common targets between these 8 miRs (Fig. 3D,E, Supplementary Fig. 5; Supplementary Tables 10–16). The brain-specific miR-124 can trigger apoptosis-inhibitory pathways by targeting pro-apoptotic BCL-2 proteins^36^. Thus, we sought for BPs overrepresented among common targets and focused on miR pairs converging on the regulation of the BP apoptosis (Fig. 3E, Supplementary Fig. 5, Supplementary Tables 10–16). Previous studies of context features present in target 3′UTRs, which influence the targeting efficacy of miR beyond base pairing within “seed” regions, have established that the proximity of sites for coexpressed miRs is an important determinant of cooperative action^24^,^37^. We applied this and other possible inclusion criteria for miR cooperativity, as follows: 1) miRs with at least a 6mer base pairing region (allowing only one G:U wobble) within the first or last quartile of the BCL2L13 3′UTR and 2) 80nt proximity between seed regions (Fig. 3F, Supplementary Fig. 5 and Supplementary Table 9). We adopted these criteria considering distance constraints between miR binding sites known to influence efficacy, and in particular cooperativity, and to include most previously characterized seed-matched binding sites and maximal amount of G:U wobbles allowed in them for translational repression^38^,^39^,^40^. Following this approach, miR-135a and miR-137 were identified as the two strongest candidates to cooperate with miR-124 in the regulation of BCL2L13 expression. While only 30 out of 633 (3.1%) common targets between miR-124 and miR-135a were linked to apoptosis, miR-124 and miR-137 shared 336 GO annotated target genes, with 61 (18, 2%) involved in the BP apoptosis, included BCL2L13 (Fig. 3D and Supplementary Tables 7–9). The upregulation of miR-124 and miR-137 was validated by RT-QPCR (Supplementary Fig. 3G,H). These results suggested a cooperative action between miR-124 and miR-137 in the regulation of apoptotic functions through BCL2L13 in NSPC. Therefore, we decided to investigate further a possible cooperativity between these two miRs on the regulation of BCL2L13.

### BCL2L13 expression in intermediate neuronal progenitors of the DG and in primary post-natal hippocampal NSPC cultures

**Figure 4:**
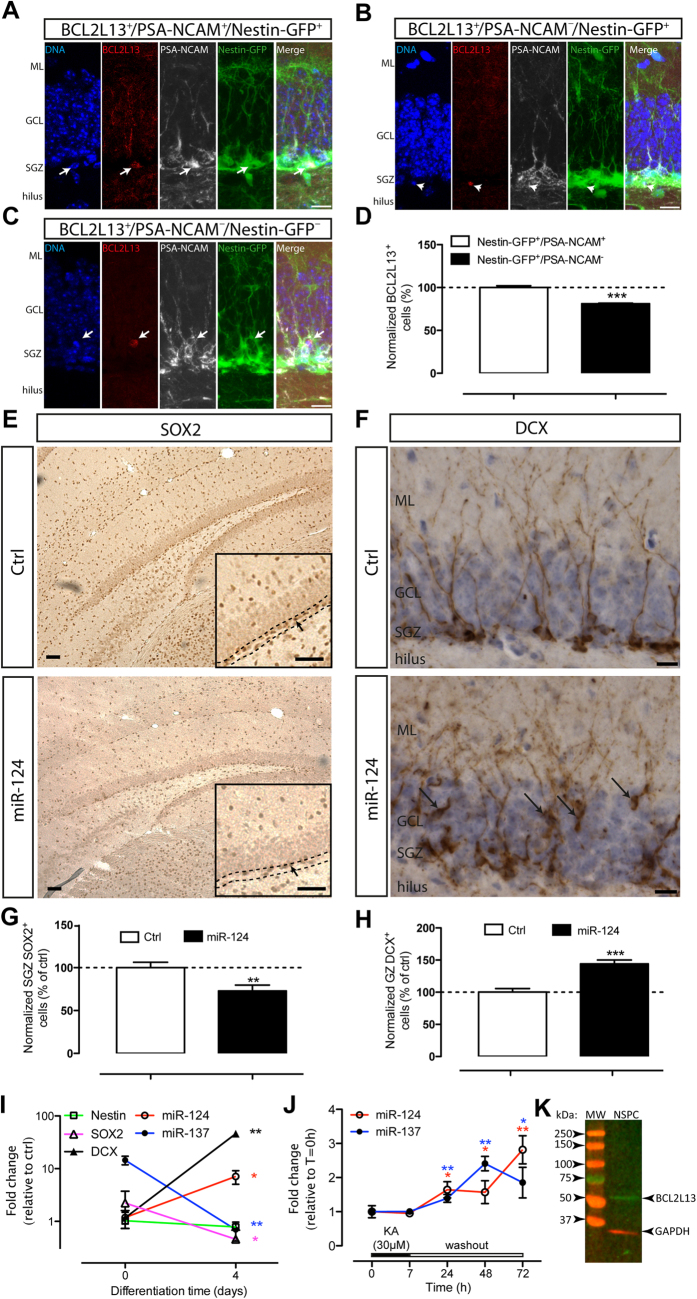
BCL2L13 expression in the DG and hippocampal NSPC cultures. (**A**) Representative confocal image displaying BCL2L13+/PSA-NCAM+/Nestin-GFP + cells present in the SGZ (arrows). (**B**) Representative confocal image displaying BCL2L13+/PSA-NCAM-/Nestin-GFP + cells present in the SGZ (arrowheads). (**C**) Representative confocal image displaying BCL2L13+/PSA-NCAM-/Nestin-GFP- cells outside the SGZ (arrows). (**D**) Quantification of BCL2L13 protein expression in Nestin-GFP + cell populations of the DG (***p < 0.001). (**E**) SOX2 + cells 7 days after infusion with a NT-miR (Ctrl, top) or miR-124 (bottom). Insets: black arrowheads indicate SOX2 + cells within the SGZ, dotted lines indicate the limits of the SGZ. (**F**) DCX + cells 7 days after infusion with a NT-miR (Ctrl, top) or miR-124 (bottom). Black arrows: ectopic DCX + cells in the GCL. (**G**) Quantification of SOX2 + cells in the SGZ 7dpi with a NT-miR (Ctrl) or miR-124 (**p < 0.01). (**H**) Quantification of DCX + cells in the GZ 7dpi with a NT-miR (Ctrl) or miR-124 (***p < 0.001). (**I**) RNA expression of cell markers and miRs in hippocampal NSPC cultures, (SOX2, *p < 0.05; miR-137, **p < 0.01; DCX, **p < 0.01; miR-124, *p < 0.05; Nestin, *p* > 0.05). (**J**) miR-124 and miR-137 expression in hippocampal NSPC cultures during and after treatment with 30 μM KA, (*p < 0.05 and **p < 0.01, relative to time = 0). **(K)** BCL2L13 protein expression in hippocampal NSPC cultures. Left lane: molecular weight marker (MW), right: BCL2L13 and GAPDH. Data in bar graphs represent mean normalized numbers of immunoreactive cells (% of Nestin-GFP + /PSA-NCAM + or Ctrl) ± SEM from three animals. ML: molecular layer, GCL granular cell layer, SGZ: subgranular zone, GZ: granular zone containing areas GCL + SGZ, dpi: days post injection. Scale bars represent 10 μm (**A–C** and **F**) or 50 μm (**E**).

The BCL2L13 expression pattern in the DG has not been characterized before. To this aim we used Nestin-GFP transgenic mice, in which GFP expression marks NSPC in the DG^41^. We found that BCL2L13 was expressed in Nestin-GFP + cells in the DG, and preferentially in a Nestin-GFP+/polysialic acid form of neural cell adhesion molecule (PSA-NCAM) + subpopulation (Fig. 4A,B). Only few BCL2L13 + cells were Nestin-GFP-/PSA-NCAM- and these were outside the SGZ (Fig. 4C). Nestin-GFP+/PSA-NCAM + cells are classified as intermediate neuronal progenitors^42^ and a similar cell type is affected by KA-SE^13^. MiR-124 and miR-137 are expressed in NSPC and in the adult DG and control their maturation and fate^43^,^44^,^45^. In agreement with this, 7 days after miR-124 infusion to the DG, we observed a significant reduction of sex-determining region Y-box 2 positive (SOX2) + and an increase in DCX + cells in the SGZ (Fig. 4E–H), with a marked dispersion of DCX + cells into the granule cell layer (Fig. 4F). This latter cellular phenotype strongly reflected the alterations observed shortly after KA-SE in the DG (Supplementary Fig. 1). Supporting our observations *in vivo*, we found detectable levels of endogenous miR-124, miR-137 and BCL2L13 in hippocampal NSPC cultures (Fig. 4I,K). The levels of miR-124 and miR-137 changed significantly in cells primed into differentiation (Fig. 4I), suggesting dynamic target regulation in NSPC. We did not observe significant changes in the endogenous levels of mir-124 and mir-137 in cells treated with 30 μM KA for 7h (Fig. 4J), the experimental condition we used across all our experiments *in vitro* with NSPC and KA, and previously used by others to model the effects of KA on NSPC *in vitro*^46^. However, we observed a significant increase in the endogenous levels of miR-124 and miR-137 starting 24 h after KA induction and still present 72 h after (Fig. 4J), reflecting our observations *in vivo*.

### Regulation of BCL2L13 expression and casp3 activity by miR-124 and mir-137 in hippocampal NSPC *in vivo*

**Figure 5:**
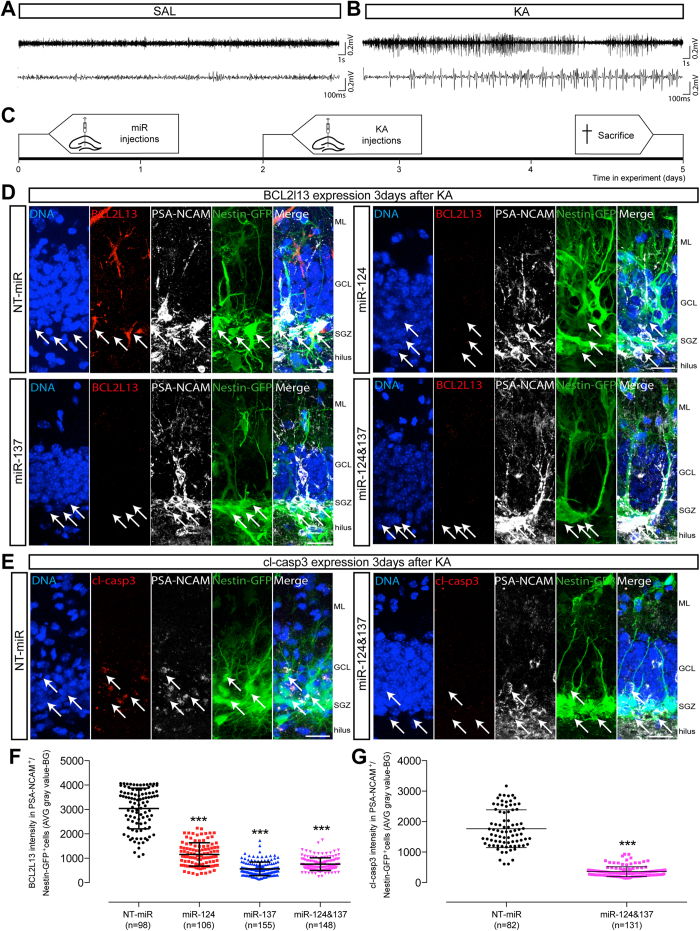
Regulation of BCL2L13 expression and casp3 activity by miR-124 and mir-137 in hippocampal NSPC *in vivo* after mild SE induced by intrahippocampal KA injection. (**A**) Representative low (top) and high (bottom) magnifications of cortical EEG recordings from freely moving animals 2 h after intrahippocampal SAL injections. (**B**) Representative low (top) and high (bottom) magnifications of cortical EEG recordings from freely moving animals 2 h after intrahippocampal KA injections, depicting peak epileptoform activity. (**C**) Schematic timeline of the experiment. Nestin-GFP mice received miR (1 μl, 50 μM total) intrahippocampal injections on day 0, intrahippocampal KA injections on day 2 and were sacrificed 3 days later, on day 5. (**D**) Representative confocal images of animals injected with miR + KA. Top left: Nestin-GFP+/PSA-NCAM+/BCL2L13 + cells (arrows) in the SGZ of mice that received NT-miR and KA. Top right: Nestin-GFP+/PSA-NCAM+/BCL2L13 + cells (arrows) in the SGZ of mice that received miR-124 + KA. Bottom left: Nestin-GFP+/PSA-NCAM+/BCL2L13 + cells (arrows) in the SGZ of mice that received miR-137 + KA. Bottom right: Nestin-GFP+/PSA-NCAM+/BCL2L13 + cells (arrows) in the SGZ of mice that received miR-124&137 + KA. (**E**) Representative confocal images of animals injected with miR and KA. Left: Nestin-GFP+/PSA-NCAM+/cl-casp3 + cells (arrows) in the SGZ of mice that received NT-miR and KA. Right: Nestin-GFP+/PSA-NCAM+/cl-casp3 + cells (arrows) in the SGZ of mice that received miR-124&137 + KA. Scale bars: 20 μm (**D** and **E**). ML: molecular layer, GCL granular cell layer, SGZ: subgranular zone. (**F**) Dot plot showing BCL2L13 expression quantified as intensities calculated from background subtracted average gray values in individual Nestin-GFP+/PSA-NCAM + cells (miR-124, ***p < 0.001; miR-137, ***p < 0.001; miR-124&137, ***p < 0.001, relative to NT-miR). (**G**) Dot plot showing cl-casp3 expression quantified as intensities calculated from background subtracted average gray values in individual Nestin-GFP+/PSA-NCAM + cells (miR-124&137, ***p < 0.001, relative to NT-miR). Values represent mean background (BG) subtracted average (AVG) gray value ± SD of at least 3 animals (**F** and **G**).

The expression of BCL2L13 in intermediate neuronal progenitors of the DG suggested a function in the regulation of casp3 activity in this cell type in the context of KA-induced hyperactivation and a possible modulation of this by miR-124 and/or miR-137. To test this hypothesis we first infused miR-124, miR-137 or an equimolar combination of both to the DG of Nestin-GFP transgenic mice (Supplementary Material and Methods). 2 days after miR infusion, mice were exposed to a mild SE induced by intrahippocampal injection of KA, which promotes neurogenesis^47^ as is the case in our systemic KA injection model (Supplementary Figure 1) and analyzed its consequences on BCL2L13 and casp3 activation in intermediate neuronal progenitors *in vivo* 3 days after SE. In our hands, saline-injected controls (SAL) did not display seizures or abnormal trains of spike activity in their EEG recordings at any time during the monitoring (Fig. 5A). However, infusion of 50 nL of 2.22 mM KA to the DG resulted in a mild SE, characterized by single and brief repetitive trains of spike activity as shown in cortical EEG traces obtained from EEG recordings sampled at 500 Hz from freely moving mice 2 h after KA administration (Fig. 5B), in agreement with recent observations using similar techniques^47^. Three days after this mild SE, we observed a significant reduction in the expression of BCL2L13 in Nestin-GFP+/PSA-NCAM + cells of the DG in mice infused with miR-124, miR-137 or an equimolar combination of both (Fig. 5D,F). Interestingly, this miR-induced reduction in BCL2L13 correlated with a significant reduction in activated casp3 expression in the same Nestin-GFP+/PSA-NCAM + cell type in animals infused with the equimolar combination of both miRs (Fig. 5E,G). These results suggest a regulation of BCL2L13 expression and casp3 activity in intermediate neuronal progenitors in the DG in the context of KA-induced hyperactivation through a possible cooperative action between miR-124 and miR-137, which we decided to characterize further *in vitro*.

### Regulation of BCL2L13 by cooperative action of miR-124 and miR-137

**Figure 6:**
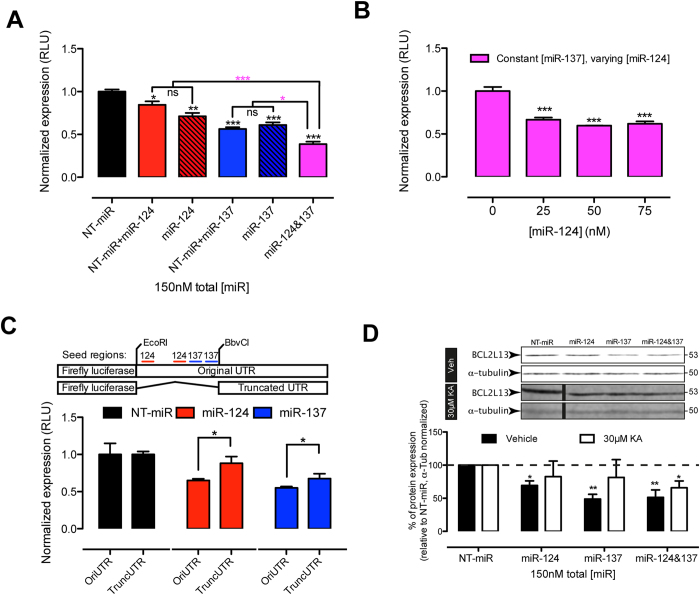
Validation of BCL2L13 as a miR-124 and miR-137 cooperative action. (**A**) Effect of miR-124 and miR-137 alone or in combination on BCL2L13 3′UTR-driven luciferase expression. Red empty bar: 75 nM miR-124 + 75 nM NT-miR; *p < 0.05; red dashed bar: 150 nM miR-124; **p < 0.01; (blue empty bar: 75 nM miR-137 + 75 nM NT-miR; ***p < 0.001; blue dashed bar: 150 nM miR-137, ***p < 0.001; (purple bar: 75 nM miR-124 + 75 nM miR-137; ***p < 0.001. All conditions compared to 150 nM NT-miR (black bar). The equimolar combination of miR-124 and 137 (purple bar) induced significantly larger downregulation of luciferase, compared to all other conditions (red empty bar: ***p < 0.001; red dashed bar, ***p < 0.001; blue empty bar, *p < 0.05; blue dashed bar, *p < 0.05). Values represent mean normalized expression (RLU) ± SEM of three independent experiments. (**B**) Effects of increasing concentrations of miR-124 in the presence of 75 nM miR-137. Values represent mean normalized expression to 75 nM miR137 + 75 nM NT-miR (0 nM miR-124) ± SEM of three independent experiments (***p < 0.001 compared to 0 nM miR-124). (**C**) Scheme of original and truncated pEZX-MT01-mouse-3 kb-BCL2L13-3′UTR and bar graph showing a significant reduction of luciferase expression (OriUTR + NTmiR vs. + miR-124 or + miR-137, *p < 0.05) and a significant rescue (Ori. vs. Trunc. UTR with same miR, *p < 0.05) of miR-mediated luciferase expression in the absence of miR-124 and 137 binding regions. Values represent mean normalized expression (RLU) ± SEM of three independent experiments. In all cases, total miR concentration was kept constant at 150 nM by adding non-targeting miR (NT-miR). (**D**) Representative immunoblots and bar graph displaying miR induced changes in endogenous BCL2L13 expression in hippocampal NSPC. Black bars: vehicle-treated NSPC cultures, 150 nM miR-124 (miR-124, *p < 0.05), 150 nM miR-137 (miR-137 **p < 0.01) and 75 nM miR-124 + 75 nM miR-137 (miR-124&137, **p < 0.01) significantly reduced BCL2L13 protein expression. White bars: KA-treated NSPC cultures, only 75 nM miR-124 + 75 nM miR-137 significantly reduced BCL2L13 protein expression (miR-124&137, *p < 0.05). All miR treatments compare to 150 nM NT-miR. Values represent mean ± SEM of three independent experiments performed in triplicates. Bands belong to the same blot, but were re-ordered for clarity of the figure. Cropping lines are indicated by vertical black lines and full-length blots are presented in Supplementary Fig. 7. In all cases total miR concentration was kept constant at 150 nM by adding NT-miR.

Using a Luciferase-BCL2L13 3′UTR reporter construct (pEZX-MT01-mouse-3 kb-BCL2L13-3′UTR), we validated BCL2L13 as miR-124 and miR-137 target. Cotransfection of HeLa cells with the reporter construct and miR-124 or miR-137 resulted in a significant reduction in luciferase expression (Fig. 6A). Increasing miR-124 and miR-137 concentrations beyond 75 and up to 150 nM did not further increase luciferase downregulation under these experimental conditions. Strikingly, an equimolar combination of miR-124 and miR-137 (75 nM each) induced the largest decrease in luciferase expression (Fig. 6A). Furthermore, miR-124 concentrations as low as 25 nM significantly downregulated luciferase expression only in the presence of miR-137 (Fig. 6B). Next, we followed a 3’UTR truncation approach, comparable to that recently used by others to study the cooperative action of miRs^48^. This truncation removed a 1.3 kb fragment of BCL2L13’s 3′UTR containing all predicted miR-124 and miR-137 binding regions and their proximal 3′UTR context as well, which may be of relevance for miR cooperativity. Removal of this fragment resulted in a significant, albeit not complete, rescue of luciferase downregulation induced by miR-124 and miR-137 (Fig. 6C). These observations point at BCL2L13 as a target of miR-124 and miR-137. Further, the marked effect observed in the presence of both miRs and the potentiation of miR-124-induced downregulation of BCL2L13 3′UTR-driven luciferase by miR-137 support the hypothesis of miR cooperativity.

### Regulation of endogenous BCL2L13 expression by the cooperative action of miR-124 and miR-137 in NSPC exposed to KA

Previous studies have demonstrated that NSPC are responsive to KA *in vitro*^46^,^49^. Following a similar experimental approach, hippocampal NSPC cultures were incubated with vehicle or 30 μM KA for 7 h. In vehicle treated cells either miR-124 or miR-137 at 150 nM or in equimolar combination (75 nM each) downregulated endogenous BCL2L13 protein levels as compared to NT-miR (Fig. 6D). In cells exposed to KA, miR-124 or miR-137 alone (150 nM) failed to induce significant BCL2L13 downregulation. However, cotransfection with an equimolar (75 nM each) combination downregulated endogenous BCL2L13 protein levels (Fig. 6D). Thus, these observations, together with those presented in Fig. 4J, suggest that the lack of effect of miR-124 and miR-137 individually on BCL2L13 expression may not be simply explained by an induction of endogenous miR levels, and indicate that miR-124 and miR-137 cooperative action on BCL2L13 expression may be a relevant regulatory mechanism in NSPC exposed to KA.

### Effect of BCL2L13 overexpression on CytC release and casp3 activation in NSPC exposed to KA

**Figure 7:**
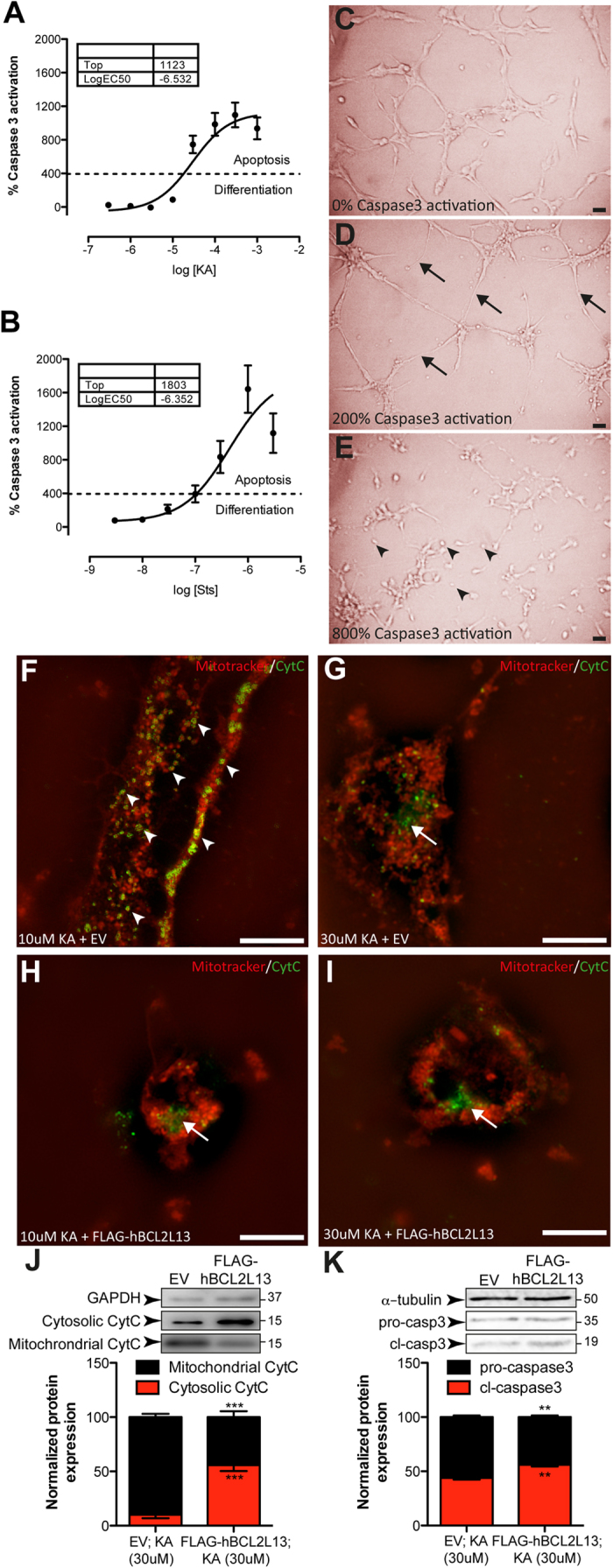
Effect of KA and exogenous BCL2L13 expression on CytC release from mitochondria and casp3 activation in hippocampal NSPC cultures. (**A**) Treatment with increasing concentrations of KA. Values are expressed as casp3 activity normalized to vehicle treated cells. (**B**) Treatment with increasing concentrations of Sts. Values are expressed as casp3 activity normalized to vehicle treated cells. Dashed lines represent the transition from differentiation to apoptosis. (**C**) Cell morphology under vehicle treatment conditions. (**D**) Cell morphology after treatment with 10 μM KA, inducing low casp3 activity (200% increase) as shown in (**B**). Arrows: cells displaying thinning and neurite extension. (**E**) Cell morphology after treatment with 30 μM KA, inducing higher amounts of casp3 activity (800% increase) as shown in (**B**). Arrowheads: shrunk cells. (**F**) SIM image showing details of hippocampal NSPC after 10 μM KA treatment. Arrowheads: CytC immunoreactivity in Mitotracker + mitochondria (yellow). (**G**) SIM image showing details of hippocampal NSPC after 30 μM KA treatment. Arrows: CytC immunoreactivity outside Mitotracker + mitochondria (green). (**H**) SIM Image showing details of hippocampal NSPC transfected with FLAG-hBCL2L13 after 10 μM KA treatment. White arrow points to CytC immunoreactivity outside Mitotracker + mitochondria (green). (**I**) Representative SIM micrograph showing hippocampal NSPC transfected with FLAG-hBCL2L13 after 30 μM KA treatment. Arrow: CytC immunoreactivity outside Mitotracker + mitochondria (green). Cells shown in (**F** and **G**) were transfected with EV for comparison to FLAG-hBCL2L13 (**H** and **I**). (**J**) Effect of 30 μM KA treatment in combination with EV or FLAG-hBCL2L13 on CytC localization. FLAG-hBCL2L13 transfection decreased the ratio of mitochondrial to cytosolic CytC expression significantly, compared to EV transfection (***p < 0.001). (**K**) Effect of 30 μM KA treatment in combination with EV or FLAG-hBCL2L13 transfection on casp3 activation. FLAG-hBCL2L13 transfection decreased the pro-casp3/cl-casp3 ratio significantly, compared to EV transfection (**p < 0.01). Scale bars: 10 μm (**C–E**); 3 μm (**F–I**). Values represent normalized mean (% of vehicle or EV) ± SEM of three independent experiments performed in triplicates.

Hippocampal NSPC cultures were treated with increasing concentrations of KA, and morphological alterations were recorded prior to casp3 activity measurements. Low KA concentrations induced low levels of casp3 activation, paralleled by neurite extension, a morphological change associated with NSPC differentiation (Fig. 7A,D). High KA concentrations induced higher levels of casp3 activity paralleled by cell shrinkage, a morphological change associated with apoptosis (Fig. 7A,E). Likewise, high concentrations of KA reduced CytC localization to mitochondria (Fig. 7F–I), suggesting CytC release associated with the permeabilization of the mitochondrial outer membrane. Vehicle treated cells were used as reference to identify morphological changes induced by KA (Fig. 7C). The effects of KA on casp3 activity were mimicked by Staurosporine (Sts), a potent activator of casp3, cellular differentiation and apoptosis in NSPC^50^ (Fig. 7B).

Transfections of exogenous BCL2L13 (validated in Supplementary Figure 6A) aggravated KA-induced CytC release (Fig. 7H,J) and casp3 activation (Fig. 7K). Furthermore, treatment of NSPC with specific siRNAs to knockdown BCL2L13 resulted in a significant downregulation of BCL2L13 protein and a concomitant significant decrease in casp3 activation in NSPC cells treated with KA (Supplementary Figure 6B,C). Overall these results suggest that the control of BCL2L13 protein levels is relevant for CytC release from mitochondria and casp3 activation in NSPC exposed to KA.

### Functional relevance of the cooperative regulation of BCL2L13 by miR-124 and miR-137

**Figure 8:**
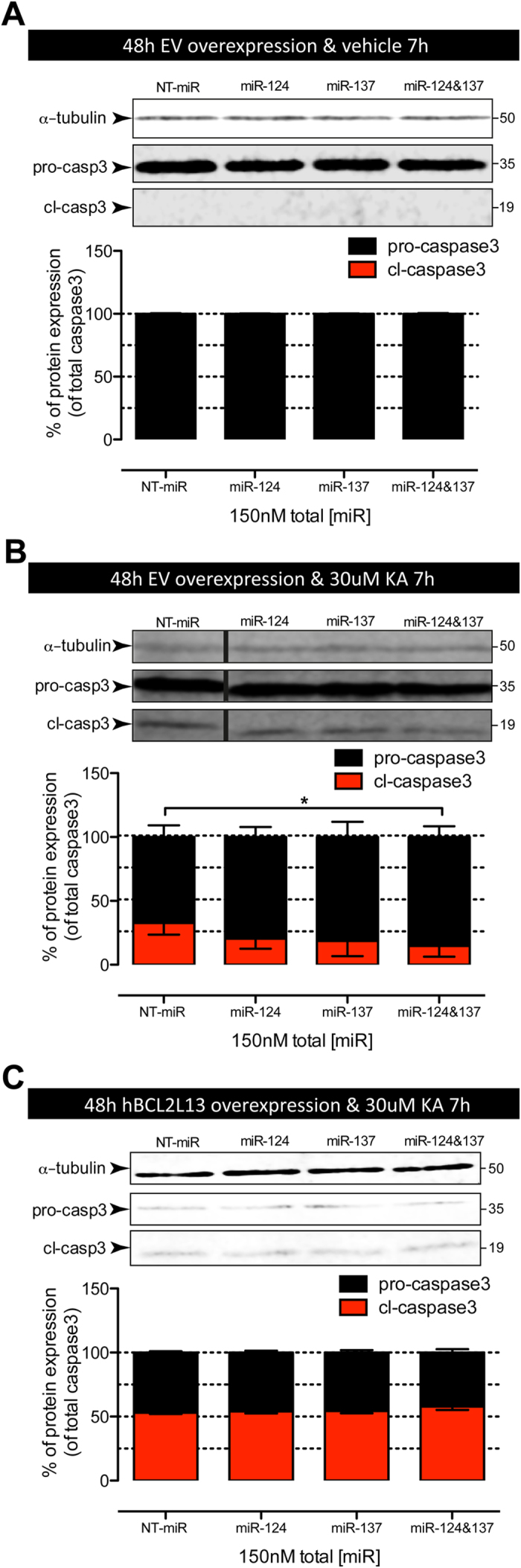
Effect of miR-124 and miR-137 alone or in combination on endogenous BCL2L13 and casp3 activation in hippocampal NSPC cultures. (**A**) Transfection with 150 nM miR-124 (miR-124), 150 nM miR-137 (miR-137) or 75 nM miR-124 + 75 nM miR-137 (miR-124&137) did not result in significant changes in pro-casp3 expression levels in vehicle-treated cultures (*p* > 0.05, compared to NT-miR). Cl-casp3 expression was below detection levels. (**B**) Transfection with 75 nM miR-124 + 75 nM miR-137 resulted in a significant reduction in cl-casp3 levels after 30 μM KA treatment (*p < 0.05, compared to NT-miR). All other miR transfections led to non-significant differences. All bands belong to the same blot, but were re-ordered for clarity of the figure. Cropping lines are indicated by vertical black lines and full-length blots are presented in Supplementary Fig. 7. (**C**) Effect of 75 nM miR-124 + 75 nM miR-137 on cl-casp3 levels was abolished by co-transfection with a BCL2L13 construct devoid of its 3′UTR. There were no significant differences between transfections with 150 nM miR-124 (miR-124), 150 nM miR-137 (miR-137) or 75 nM miR-124 + 7  nM miR-137 (miR-124&137), *p* > 0.05, compared to 150 nM NT-miR. Values represent mean ± SEM of three independent experiments performed in triplicates. In all cases total miR concentrations were kept constant at 150 nM adding NT-miR.

Finally, we investigated the effects of miR-124 and miR-137 on the expression of active, cleaved casp3 (cl-casp3) in hippocampal NSPC cultures exposed to KA. In vehicle-treated cells, neither miR-124, miR-137, nor the combination of both had detectable effects on uncleaved casp3 (pro-casp3) or cl-casp3 expression levels (Fig. 8A). However, in cells incubated with KA, co-transfection with an equimolar (75 nM) combination of miR-124 and miR-137 reduced cl-casp3 expression. Neither miR-124 nor miR-137 alone (150 nM) were able to induce changes in cl-casp3 levels (Fig. 8B). Transfection with a FLAG-BCL2L13 construct devoid of its 3′UTR reverted cl-casp3 downregulation induced by the equimolar combination of miRs (Fig. 8C). These results indicate that miR-124 and miR-137 cooperativity regulates BCL2L13 protein levels and controls casp3 activity in NSPC exposed to KA.

## Discussion

Here, we investigated relationships between changes in gene expression induced in the DG after KA-SE and alterations linked to mitochondrial function. We show that: 1) a group of 114 proteins is differentially expressed in the DG 3 days after KA-SE; 2) these proteins can be categorized into overrepresented networks and biological functions, including mitochondria-dependent apoptosis; 3) within a group of significantly downregulated proteins linked to mitochondrial function, we identified the BCL-2 protein BCL2L13. BCL2L13 controls CytC release and casp3 activity in hippocampal NSPC cultures; 4) BCL2L13 is a hitherto uncharacterized miR-124 and miR-137 target, regulated by the cooperative action of both miRs and 5) the cooperative action of miR-124 and miR-137 regulates BCL2L13 protein levels and controls casp3 activity in NSPC exposed to KA. Our observations suggest that BCL2L13 controls casp3 activation, fine-tuning mitochondria-dependent apoptotic pathways in NSPC.

Previous observations have suggested that protein expression is predominantly controlled at the level of translation in mammalian cells^51^,^52^, underscoring the importance of posttranscriptional regulation. In our experiments, changes in protein levels detected by proteomics did not correlate well with changes in corresponding mRNA, thus suggesting posttranscriptional regulation. This conclusion emphasizes the advantage of including proteomics-supported miR targets in our studies. Therefore, in a primary effort to understand the contribution of posttranscriptional mechanisms, we focused on miR regulation as a possible mechanism to explain the observed discrepancies between protein and corresponding mRNAs levels. In agreement with recent observations^53^, we found more up- than down-regulated miRs, indicating they may be implicated in the control of some of the changes in gene expression taking place in the DG shortly after KA-SE. Bioinformatics predictions have identified apoptosis as a common pathway among 14 miRs upregulated in various models of SE. Importantly, miR-124 has been implicated in the inhibition of neuronal apoptosis in the DG. Deletion of Rncr3, the dominant source of miR-124a, results in a significant increase in the number of apoptotic cells in the DG without affecting NSPC proliferation, indicating that miR-124 anti-apoptotic function is essential for the maturation and survival of DG neurons^43^. Suggestively, we observed that intrahippocampal infusion of miR-124 resulted in phenotypic changes in immature cells of the DG that were reminiscent of those induced by KA-SE. Furthermore, intrahippocampal infusion of miR-124, miR-137 or a combination of both previous to KA infusion resulted in a stronger downregulation of BCL2L13 expression in NSPC in the DG, which correlated with a stronger reduction in activated casp3 when both miRs were combined, suggesting a cooperative miR action.

miR cooperativity could explain the coordinated action of multiple miRs, rendering targets more sensitive to relatively small changes in levels of individual miRs^24^, such as those described herein for brain specific or enriched miRs. Therefore, we focused on the hypothesis that miR-124 may cooperate with other upregulated brain specific or enriched miRs to regulate specific targets relevant for NSPC survival. To test this hypothesis we focused on mitochondrial BCL-2 proteins, particularly on BCL2L13, whose protein expression levels did not correlate well with corresponding mRNA levels. Following a bioinformatic pipeline in which we analyzed cooperativity between 11 upregulated miRs, common targets and converging biological pathways, we were able to narrow down the number of potential biologically relevant targets, leading to the identification of BCL2L13 as a novel target of miR-124 and miR-137 cooperativity. We identified multiple predicted binding sites for the brain specific or enriched miR-124 and miR-137 in BCL2L13′s 3′UTR and characterized BCL2L13 as a novel target of these two miRs.

To better understand the functional relevance of miR-124 and miR-137 cooperativity, we focused on the role of BCL2L13 in NSPC. We found that BCL2L13 is preferentially expressed in intermediate progenitor cells of the DG, indicating a functional role in these cells. In NSPC cultures, changes in casp3 induced by low or high KA doses were associated with phenotypic changes indicative of NSPC differentiation or apoptosis respectively, reflecting the dual role of casp3 activation in NSPC^17^. We found that miR-124 and miR-137 cooperatively regulated BCL2L13 in NSPC exposed to KA *in vivo* and *in vitro*, indicating that cooperativity between these two miRs is involved in fine-tuning the levels of apoptosis-related proteins. Underscoring the functional relevance of miR cooperativity in KA-treated NSPC, miR-124 and miR-137 did not have a significant effect on BCL2L13 protein levels or casp3 activation individually, yet they decreased BCL2L13 levels and inhibited casp3 activation when administered in combination. Moreover, exogenous expression of a miR-insensitive BCL2L13 reverted the reduction of active casp3 mediated by miR-124 and miR-137 and was associated with increased cytosolic CytC localization, leading to the possibility that miR-124 and miR-137 cooperativity fine-tunes BCL2L13 to favor non-apoptotic caspase-3 functions in NSPC. Further demonstration of this concept would require loss-of-function approaches, aimed to knowckdown or inhibit miR actions. However, these approaches are difficult to optimize experimentally and all have their advantages and disadvantages, implying that a combination of multiple approaches may be necessary to establish miR function^54^. Furthermore, results obtained with antimiR oligonucleotides might be difficult to bring into line with those obtained with miR mimics at low endogenous miR expression levels^48^, such as those we observed in NSPC cultures.

Overall, our observations may be relevant to understanding in more detail the regulation of adult hippocampal neurogenesis after SE and its possible consequences for epileptogenesis. Interestingly, recent observations have demonstrated that mitochondria play a key role in adult hippocampal neurogenesis, mitochondrial dysfunction influences NSPC differentiation^55^,^56^ and mitochondrial complex I deficiency is observed in hippocampal biopsies of TLE patients^57^, indicating that our experimental model may reflect relevant aspects of the human disorder. Although speculative at this point, BCL2L13 downregulation together with changes in CLU expression and in the mitochondrial NADH:ubiquinone oxidoreductase complex I, suggested by the downregulation of two of its subunits we observed in the DG after KA-SE, may provide a link between mitochondria dysfunction and the regulation of adult neurogenesis in early stages of epileptogenesis. Validation of this hypothesis and the potential impact of reduced BCL2L13 expression on the elimination of excessive cell numbers and network reorganization in the DG^5^,^6^ require further experiments. However, previous observations have shown that the mitochondrial apoptosis pathway is required for normal organization and function of newborn neurons in the DG^58^. These, together with our findings, suggest that fine-tuning of key components of mitochondrial apoptotic pathways, mediated by miR-124 and miR-137 cooperativity on BCL2L13 and its associated functions, may contribute to the early response to epileptic seizures in the DG.

## Materials and Methods

### Animals, SE induction and tissue collection

6–8 week-old male C57BL/6j mice were used (Janvier Bioservices, Genest st Isle, France). Mice were housed in groups for one week under a 12-hour dark/light cycle (lights on at 6.30 h) in a temperature- and humidity-controlled room, with free access to food and water. Animal experiments were approved by the committee of Animal Health and Care, Leiden University (Protocol #08170) and were performed in accordance with the guidelines and regulations of the European Union for the use of animals for scientific purposes. Mice randomly assigned to experimental groups were injected with Kainic acid (KA; Sigma K0250, Kainic acid monohydrate) or Vehicle (Saline; SAL, 0.9% NaCl), following a protocol of multiple, low-dose, intraperitoneal injections of KA^59^. SE was induced by repeated injections of KA (10 mg/ml in saline, pH 7.4). The starting dose was 24 mg/kg and subsequent injections of 6 mg/kg were given every 30 minutes until SE occurred^59^. Behavioural seizures were scored after each KA injection using a modified Racine’s scale^60^. Only animals displaying unequivocally class IV-V seizures that lasted at least 5 minutes were selected for future experiments. Control animals were injected with equal amounts of SAL. Mice injected with KA or SAL (n = 3 per group) were sacrificed 3 days after. Brains were extracted and immediately placed in ice-cold artificial CSF (NaCl: 124; KCl: 2.5; NaH2PO4: 1.25; CaCl2: 1; MgCl2: 1; NaHCO3: 25; D-Glucose: 10; all in mM) constantly bubbled with 95%O2/5% CO2, sectioned with a vibratome and the DG was separated from other hippocampal regions along the hippocampal fissure and avoiding contamination from the third ventricle using a previously described microdissection procedure^49^. For transcriptomics and proteomics analyses, hippocampi of the same individuals were used. The right DG was reserved for genomic and the left for proteomic analyses^61^. For histological preparations brains of three additional mice were used per experimental group.

### Nano-LC peptide separation

Tissue homogenization and fractionation was carried out using gel electrophoresis and in-gel digestion. Tissue samples of microdissected DG from the KA or SAL-treated groups were lysed in lysis buffer (per 100 mg tissue, one ml buffer containing 7 M urea, 2 M thiourea, 4% (w/v) CHAPS, and 10 μl/ml protease inhibitor mix (Amersham Biosciences, Piscataway, NJ, USA)). Protein (30 μg) fractions were loaded on gradient gels (NuPAGE 4–12% Bis-Tris gel, 1 mm × 10 wells, Invitrogen). The gels were then stained with Coomassie Brilliant Blue G-250 (Pierce, Rockford, IL, USA). Before MS analysis, separated proteins were in-gel digested as previously described^62^. Further details on Nano-LC peptide separation, mass spectrometry, protein identification and quantification are described in Supplementary Data.

### Microarray gene expression profiling

RNA extraction, sample preparation, hybridization to microarray, and detection were performed as described before^63^. Further details are described in Supplementary Data.

### miR expression profiling

The same total RNA samples quality-controlled and used for gene expression profiling were used to profile 381 mature mouse miRs using 384-well TaqMan Array Mouse MicroRNA Fluidic v3.0 Cards, in triplicates (Applied Biosystems, Life Technologies), following a method previously used for to the identification of miR expression profiles in rat brain and blood after KA-SE^21^. The U6 small nuclear RNA was used as endogenous control and an unrelated human total RNA sample was included as a negative control. Samples were processed and analyzed by an investigator blinded to treatment using a previously described method^64^. Further details are described in Supplementary Data.

### In silico cooperative miR target prediction analysis

The miRecords database, composed of 11 established miR target prediction programs^65^ was used to produce a list of mRNA targets for a group of previously characterized brain- enriched or -specific miRs^35^, significantly regulated in our experiments. Further details are described in Supplementary Data.

### NSPC culturing and transfections

Primary post-natal hippocampal NSPC cultures were prepared, cultured and transfected as previously described^66^. Further details are described in Supplementary Data.

### Western blotting

Western blotting was performed as previously described^66^. Further details are described in the Supplementary Data.

### 3′UTR Luciferase reporter assays

miRs were tested for functional knockdown efficiency with a dual luciferase reporter assay following manufacturers instruction (Dual-Luciferase Reporter Assay System, Promega), luciferase activity was measured in cell lysates using a Spectramax L luminometer, as described before^67^. Further details are described in Supplementary Data.

### Quantitative real time polymerase chain reactions

mRNA and miR QPCRs were performed as described before^67^. Further details are described in Supplementary Data.

### Stereotactic miR infusion, immunohistochemistry and confocal microscopy

Stereotactic surgery was performed as described before^66^ and was approved by the committee of Animal Health and Care, University of Amsterdam (DEC#236 and DEC#314) and were performed in accordance with the guidelines and regulations of the European Union for the use of animals for scientific purposes. Further details are described in Supplementary Data.

### Electrode implantation, EEG recording and electrophysiological characterization

Stereotactic surgery was performed as described before^66^ and was approved by the committee of Animal Health and Care, University of Amsterdam (DEC#296) and were performed in accordance with the guidelines and regulations of the European Union for the use of animals for scientific purposes. Further details are described in Supplementary Data.

### Casp-3 activity

Casp-3 activity was measured using a Caspase 3 Fluorimetric Assay Kit (Cat# CASP3F, Sigma-Aldrich), as previously described^63^. Further details are described in Supplementary Data.

### Immunocytochemistry, mitochondrial staining and structured illumination microscopy (SIM)

50 thousand hippocampal NSPC were seeded per well in 24-well plates containing poly-L-lysine and Laminin coated glass coverslips as described before^68^. The next day cells were transfected with FLAG-tagged human BCL2L13, a kind gift from Dr. Jürg Tschopp, Institute of Biochemistry, University of Lausanne (Kataoka *et al.*, 2001), using Attractene (Qiagen) or empty vector and were incubated for 48 h. In the last 7 h of the incubation cells were treated with varying concentrations of KA ranging from 0–300 μM KA or vehicle^46^. Cells were treated with 250 nM MitoTracker® Red CMXRos (Invitrogen) for 5 minutes to specifically stain mitochondria^69^ and fixed for 15 minutes in 4% PFA. Subsequently, cells were stained with polyclonal sheep anti-CytC (Sigma-Aldrich, 1:100) in combination with donkey anti-sheep Alexa488 (Invitrogen, 1:1000) and coverslips were mounted in vectashield mounting medium (Vector Labs). SIM was performed using a Nikon Eclipse Ti inverted microscope based SIM system as described before^68^.

### Statistical analysis

All comparisons were statistically tested using unpaired two-tailed Student’s *t*-test. When more than two groups were compared, one-way analysis of variance (ANOVA) test with Tukey’s post test was used. For correlative relations Pearson’s correlation analysis was performed. All statistical analyses were performed using GraphPad Prism 5.0.

## Additional Information

**How to cite this article**: Schouten, M. *et al.* MicroRNA-124 and -137 cooperativity controls caspase-3 activity through BCL2L13 in hippocampal neural stem cells. *Sci. Rep.* **5**, 12448; doi: 10.1038/srep12448 (2015).

## Supplementary Material

Supplementary Information

Supplementary Figure 1

Supplementary Figure 2

Supplementary Figure 3

Supplementary Figure 4

Supplementary Figure 5

Supplementary Figure 6

Supplementary Figure 7

Supplementary Table 1

Supplementary Table 2

Supplementary Table 3

Supplementary Table 4

Supplementary Table 5

Supplementary Table 6

Supplementary Table 7

Supplementary Table 8

Supplementary Table 9

Supplementary Table 10

Supplementary Table 11

Supplementary Table 12

Supplementary Table 13

Supplementary Table 14

Supplementary Table 15

Supplementary Table 16
