# Supplementary material for: MicroRNA-124 and -137 cooperativity controls caspase-3 activity through BCL2L13 in hippocampal neural stem cells
Source: Sci Rep. 2015 Jul 24;5:12448. doi: 10.1038/srep12448 (PMC4513647; doi:10.1038/srep12448)
Supplement: Supplementary Information [file srep12448-s1.doc]

**Supplementary data to:**

MicroRNA-124 and -137 cooperativity controls caspase-3 activity through BCL2L13 in hippocampal neural stem cells

**Authors**

Marijn Schouten^1^, Silvina A. Fratantoni^2^, Chantal J. Hubens^3, 4, #^, Sander R. Piersma^2^, Thang V. Pham^2^, Pascal Bielefeld^1^, Rob A. Voskuyl^3, 4^, Paul J. Lucassen^1^, Connie R. Jimenez^2^ & Carlos P. Fitzsimons^1, *^.

**Affiliations**

1 Center for Neuroscience, Swammerdam Institute for Life Sciences, University of Amsterdam, SciencePark 904, 1098XH, Amsterdam, The Netherlands.

2 Oncoproteomics Laboratory, Cancer Center, Free University Amsterdam, De Boelelaan 1117, 1081HV, Amsterdam, The Netherlands.

3 Division of Pharmacology, LACDR, Leiden University, Einsteinweg 55, 2333 CC Leiden, The Netherlands.

4 Foundation of Epilepsy Institutes of The Netherlands (SEIN), Achterweg 5, 2103 SW, Heemstede, The Netherlands.

**Supplementary materials and methods**

**Nano-LC peptide separation**

Gels were washed and dehydrated once in 50 mM ammonium bicarbonate (ABC) and twice in 50 mM ABC/50% acetonitrile (ACN). Cysteine bonds were reduced by incubation with 10 mM DTT/50 mM ABC at 56 °C for 1 h and alkylated with 50 mM iodoacetamide/50 mM ABC at room temperature (RT) in the dark for 45 min. After washing sequentially with ABC and ABC/50% ACN, the whole gel was sliced in 10 bands of equal width for each lane. Gel bands were sliced up into approximately 1-mm^3^ cubes and collected in tubes, washed in ABC/ACN and dried in a vacuum centrifuge. Gel cubes were incubated overnight at 23 °C with 6.25 ng/mL trypsin and covered with ABC to allow digestion. Peptides were extracted once in 1% formic acid and twice in 5% formic acid/50% ACN. The volume of the peptide extract was reduced to 60 μL in a vacuum centrifuge and filtered using a 0.45 μm filter to remove gel particles and contaminants prior to LC–MS analysis. Subsequently, peptides were separated by an Ultimate 3000 nanoLC system (Dionex LC-Packings, Amsterdam, The Netherlands) equipped with a 20 cm × 75 µm ID fused silica column custom packed with 3 µm 120 Å ReproSil Pur C18 aqua (Dr Maisch GMBH, Ammerbuch-Entringen, Germany). After injection, peptides were trapped at 6 µl/minute in 1.6% acetonitrile + 0.05% formic acid on a 1 cm × 100 µm ID precolumn packed with 5 µm ReproSil Pur C18 aqua. Peptides were separated in a 60 minutes gradient (8-32% acetonitrile in 0.05% formic acid) at 300 nl/min. followed by washing (72% acetonitrile in 0.05% formic acid) and equilibration (4% acetonitrile in 0.05% formic acid). The inject-to-inject time was 90 minutes.

**Mass spectrometry**

Intact peptide MS spectra and MS/MS spectra were acquired on a LTQ-FT hybrid mass spectrometer (Thermo Fisher, Bremen, Germany) as described before ^1,2^ Intact masses were measured at 50.000 resolution in the ICR cell. In parallel, following an FT pre-scan, the top5 peptide signals (charge-states 2+ and higher) were submitted to MS/MS in the linear ion trap (3 amu isolation width, 30 ms activation, 35% normalized activation energy, Q value of 0.25 and a threshold of 5000 counts). Dynamic exclusion was applied with a repeat count of 1 and an exclusion time of 30 sec.

**Protein identification and quantification**

MS/MS spectra were searched against IPI mouse database 3.59 (56692 entries) using Sequest (version 27, rev 12) with a maximum allowed deviation of 10 ppm for the precursor mass and 1 amu for fragment masses. Methionine oxidation and cysteine carboxamidomethylation were allowed as variable modifications, two missed cleavages were allowed. Scaffold 2.06.01 (Proteome software, Portland, OR) was used to organize the gel-slice data and to validate peptide and protein identifications. Identifications with a Peptide Prophet probability> 95% were retained. Subsequently, protein identifications with a ProteinProphet probability of >99% with 2 peptides or more in at least one of the samples were retained. Proteins that contained similar peptides and could not be differentiated based on MS/MS analysis alone were grouped. For quantitative protein analysis across samples, spectral counts (number of identified MS/MS spectra for each protein) were normalized on the sum of the spectral counts per biological sample. Differential analysis of samples was performed using the BetaBinominal test as described previously ^3^. Protein identification and quantification details can be found in ^2,3^. Ingenuity Pathway Analysis (IPA) were performed as previously described ^3,4^.

**Microarray gene expression profiling**

Total RNA samples extracted from the hippocampi of each animal of the KA or SAL groups (n=3 per group) using TRIzol reagent (Invitrogen), were checked for their quality and integrity using Nano Lab-on-Chip technology and an Agilent Bioanalyzer. The Illumina TotalPrep RNA Amplification kit (Ambion, Life Technologies) was used to synthesize biotine-labelled cRNA and concentrations of biotinylated cRNA were measured using a Nanodrop spectrometer. A total of 1.5 µl of each biotinylated cRNA sample was hybridized onto a MouseWG-6 Expression BeadChip (Illumina) and BeadChips were scanned with the Illumina BeadArray. Gene Expression Analysis was done with Illumina’s Genome Studio software, using default settings suggested by the manufacturer. Transcript signals were subjected to quantile normalization, using the R Bioconductor ^5^package Abarray. Microarray data analyses were performed with the software packages Abarray, BRB Array Tools (Biometric Research Branch of the US National Cancer Institute, Bethesda, MD, USA; http://linus.nci.nih.gov/BRBArrayTools.html) and Spotﬁre Decisionsite (Spotﬁre, Somerville, MA, USA) as previously described ^6^. A permutation p-value cutoff < 0.01 was used as inclusion criterion.

**miR expression profiling**

Total RNA (800ng/sample) was converted into cDNA using Megaplex RT Primers (Applied Biosystems) and a TaqMan miRNA RT Kit (Applied Biosystems). cDNAs were mixed with TaqMan Universal PCR Master Mix (Applied Biosystems) and then loaded on Mouse MicroRNA Fluidic v3.0 Cards (Applied Biosystems). The cards were run using a 7900HT real-time PCR instrument (Applied Biosystems). Statistical analyses and data classification were performed as described ^7^. Those miRs for which 95% of individual observations had a raw CT score >35 were excluded from the final data analysis. Fold change filters were applied to select miRs and a t-test with FDR (false discovery rate) of 5% correction for multiple comparisons was used to examine the significance of miRs regulated in the KA group in comparison with the SAL group. miRs with fold change equal or higher than 1.5 and *p* < 0.05 were selected for further analysis.

**In silico cooperative miR target prediction analysis**

A list of predicted mRNA targets of the significantly deregulated brain enriched or specific miRs ^8^ was made using the miRecords database ^9^, which integrates the predictions of 11 established miRNA target prediction programs, including DIANA-microT ^10^ Micro-Inspector ^11^, miRanda ^12^, miR-Target2 ^13^, mi-Target ^14^, NBmir-Tar ^15^, Pictar ^16^, PITA ^17^, RNA22 ^18^, RNA Hybrid ^19^ and Targetscan ^20^, presenting a balance between rule-based and data-driven prediction approaches, which may improve miR binding region prediction accuracy ^21^. For example, conservation across species has been an important parameter used in prediction programs, however, its relevance should be considered together with other possible target recognition parameters, since almost 30% of experimentally supported mammalian miRNA–target gene interactions in a benchmark data set were non conserved, highlighting the potential relevance of nonconserved target sites ^22-24^. Following this approach, lists of predicted targets for the 8 deregulated brain enriched or specific miRs were produced using miRecords’ Predicted Target section with a filter to display putative targets predicted by at least three prediction programs. This filter was established considering previous reports indicating that the total number of programs considered in miR target prediction influences the sensitivity-specificity tradeoff of the prediction ^22^. Target predictions for all individual programs are shown in Supplementary Table S7 (miR-124), S8 (miR-137), S11 (miR-9), S12 (miR-125a-3p), S13 (miR-125b-3p), S14 (miR-135a), S15 (miR-135b), S16 (miR-190). Subsequently, miR pairs with predicted overlapping targets were analyzed for common biological processes using the GENECODIS web-based tool, hypergeometrically testing for significantly common processes (cut-off 2 transcripts per biological process, FDR corrected p<0.1) ^25-27^. To further predict the specific miR-mRNA thermodynamic binding properties, RNA22 was used ^18^.

**NSPC culturing and transfections**

Cells were cultured in culture flasks in DMEM/F-12 medium supplemented with 5% fetal bovine serum (FBS, Atlanta Biologicals), N2 supplement, (Invitrogen), Bovine Pituitary Extract (BPE, Invitrogen), recombinant-human-EGF (20ng/mL, Sigma), and recombinant-human-FGF (10ng/mL, Sigma). For differentiation of NSPC, human-EGF and recombinant-human-FGF deprived medium was used as described before (PMID: 21811434). Hippocampal NSPCs were transfected using Attractene Transfection Reagent, following the manufacturer’s instructions (Qiagen), as described before ^28^. 48 hours after transfection cell lysates were collected for analysis. In miR transfection experiments we used Pre-miR™ miRs (Ambion, Life Technologies) designed to mimic mature endogenous miRs ^29-31^. Pre-miR™ mmu-miR-124 (mature miR sequence: UAAGGCACGCGGUGAAUGCC; Ambion, Life Technologies), mmu-miR-137-3p (mature miR sequence: UUAUUGCUUAAGAAUACGCGUAG; Ambion, Life Technologies) and Non-targeting miR (Cy3 labeled; Ambion, Life Technologies). In siRNA transfection experiments, a GeneSolution cocktail directed against mouse BCL2L13 (Mm_Bcl2l13_5, CACCCTGGAGGTGACAATAA; Mm_Bcl2l13_6, CAGTATAAATCATGAAATAA; Mm_Bcl2l13_7, TAGGATTTCACTATGAAACAA; and Mm_Bcl2l13_8, TAGAATTAATTGTTCATTCAA; Qiagen) or negative control siRNA (target sequence: AATTCTCCGAACGTGTCACGT; Qiagen) was used. FLAG-tagged human BCL2L13 or empty vector, kind gifts from Dr. Jürg Tschopp, Institute of Biochemistry, University of Lausanne ^32^, were transfected using Attractene (Qiagen) according to manufacturer’s protocol and were incubated for 48h. In the last 7h of the incubation cells were treated with vehicle/KA ^33^.

**Intrahippocampal miR and KA infusion in Nestin-GFP mice**

Briefly, 6-week old male Nestin-GFP mice underwent stereotaxic surgery, during which 1.0 µL of 50µM mirVana® miRmimic (Ambion, Life technologies) was bilaterally infused into the DG (anterior-posterior: -2.0, medial-lateral: ±2.0, dorsal-ventral: -2.0). miR-124 (miRmimic sequence: UAAGGCACGCGGUGAAUGCC), miR-137 (miRmimic sequence: UUAUUGCUUAAGAAUACGCGUAG), or an equimolar mixture of both was infused into the right DG, while NT-miR was infused into the contralateral DG, serving as internal control. 48 hours after miR infusion, animals underwent a second stereotactic surgery, during which 50nL of 2.22 mM KA (Kainic Acid Monohydrate, Sigma Aldrich K0250) was bilaterally injected into the DG, at the same coordinates used for miR infusion. 72 hours after KA infusion, animals were sacrificed by transcardial perfusion-fixation, brains were extracted and processed for immunohistochemistry as described in the corresponding section.

**Electrode implantation, EEG recording, and electrophysiological characterization**

A separate batch of 6 week old C57BL/6j mice (Harlan) were used to electrophysiologically characterize the intrahippocampal KA infusion. Directly after stereotactic KA/Saline infusion, animals were implanted with epidural gold-plated stainless steel screws. Bilateral recording electrodes were fixed in the burr holes already created for KA injection, while the reference/ground electrode was placed above the right visual cortex. Electrodes were fixed to the skull bone using dental cement (Simplex Rapid, Kemdent), and connected to a common pin connector. At the end of the surgical procedure, a wireless EEG recording device (Neurologger, TSE-systems) was connected to the pin connector, allowing 24/7 EEG recordings sampled at 500Hz from freely moving mice. Data were obtained for 72 hours post KA infusion in freely moving mice and analyzed for the occurrence of epileptiform activity and epileptiform spiking.

**Western blotting**

Cells or tissue samples were lysed in ice-cold 0.5X radioimmunoprecipitation assay (RIPA) buffer (20 mM triethanolamine, 0.14 M NaCl, 0.05% deoxycetant, 0.05% SDS, 0.05% Triton X-100) supplemented with protease inhibitors (complete Protease Inhibitor Cocktail tablets; Roche Applied Science, Penzberg, Germany). Subsequently, cell lysates were centrifuged for 30 minutes at 13.000 rpm at 4°C after which the supernatants were collected. Protein content was quantified using the BCATM Protein Assay (Pierce Biotechnology, Rockefort, IL, USA) and 25 µg of each sample, were loaded onto an SDS-PAGE gel. After electrophoresis, the samples were blotted overnight onto an Immobilon P membrane (Millipore Corp., MA, USA) and processed as described . Blots were blocked in 10 mM Tris-HCl (pH 8.0), 150 mM NaCl, and 0.05% Tween 20 containing 5% nonfat dried milk powder. Proteins of interest were subsequently detected using specific primary antibodies: BCL2L13 (polyclonal goat anti-BCL2L13; Santa Cruz, 1:200), pro- and cleaved casp-3 (polyclonal rabbit anti-caspase3; Cell Signalling, 1:1000), CytC (polyclonal sheep anti-CytC; Sigma Aldrich; 1:5000), FLAG-tag (monoclonal mouse anti-FLAG M2/DYKDDDDK; Sigma, 1:1000), BAX (polyclonal rabbit anti-BAX; Abcam, 1:1000) in combination with donkey anti-goat IRdye 800 CW (Li-Cor, 1:10000), goat anti-rabbit IRDye 680 LT (Li-Cor, 1:20000), donkey anti-sheep HRP conjugated (Life Technologies, 1:2000), goat anti-mouse IRdye 680 LT (Li-Cor, 1:10000), and goat anti-rabbit IRDye 680 LT (Li-Cor, 1:20000) respectively as secondary antibodies. Expression levels of α-tubulin (monoclonal mouse anti-α-tubulin antibody; Santa Cruz, 1:200), GAPDH (monoclonal rabbit anti-GAPDH 14C10; Cell Signalling, 1:1000) or β-actin (monoclonal mouse anti-β-actin AC-15; Sigma-Aldrich, 1:2000) in combination with goat anti-mouse IRDye 680 LT (Li-Cor, 1:10000), goat anti-rabbit IRDye 680 LT (Li-Cor, 1:10000) or goat anti-rabbit HRP conjugated (Bio-Rad, 1:3000) and goat anti-mouse IRDye 680 LT (Li-Cor, 1:10000) respectively, were used for normalization. For semi-quantitative western blot analysis, a commercially available infrared fluorescence-based method for quantification of light signal from Western blots (Li-cor Odyssey FC, LI-COR Biotechnology – GmbH, Germany) was used following the method described in Bunn and Gray, *Protocol Exchange* (2011) doi:10.1038/protex.2011.274. This system allows the capture of all data in a single exposure, with both faint and strong bands captured in the linear range of detection, as validated by the manufacturer (<http://biosupport.licor.com/docs/HartaLinearityPaper-DynamicRangePaper_1114.pdf>). We used one single exposure time for all our Western blot detections (120s), to avoid bias from subjective exposure threshold setting. This exposure time was optimized to reveal the lowest abundance bands in our experiments. No saturated pixels were observed in the quantification of any band, indicating our detection optimization has placed all bands in the linear detection range. All blots were repeated at least three times, and one representative image is shown. Bar graphs represent mean±SEM of three independent experiments.

**3’UTR Luciferase reporter assays**

The first 3kb of the mouse BCL2L13 3’UTR mRNA (NCBI Reference Sequence NM_153516.2), containing predicted miR-124 and miR-137 binding regions, was cloned into the multiple cloning site of a reporter vector (pEZX-MT01, Genecopoeia) containing the firefly Luciferase open reading frame as depicted in Figure 3F (pEZX-MT01-mouse-3kb-BCL2L13-3’UTR). HeLa cells were cotransfected with pEZX-MT01-mouse-3kb-BCL2L13-3’UTR and mature miRs and/or non-targeting homologues (Pre-miR™, Ambion, Life Technologies) using Attractene Transfection Reagent (Qiagen). Cells were lysed 48 h after transfection, and ratios between Firefly luciferase and Renilla luciferase activity were calculated and expressed as relative light units (RLU). In this cloning strategy 3 out of 4 binding regions for miR-124 and miR-137 have been effectively relocated from the first quartile of the original 5.4kb 3’UTR to the center of the truncated 3kb-long 3’UTR (pEZX-MT01-mouse-3kb-BCL2L13-3’UTR, Figure 3F). This could have disfavored miR activity, resulting in a possible underestimation of knockdown efficiency. EcoRI and BbvCI (New England Biolabs) restriction enzyme digestion and subsequent Klenow Fragment Large (New England Biolabs) blunting allowed for a religation of the truncated pEZX-MT01-mouse-3kb-BCL2L13-3’UTR, using T4 DNA Ligase treatment (Fermentas), eliminating the first 1.3kb of the sequence and generating a truncated construct containing no predicted miR-124 and miR-137 binding regions (Figure 6C). Results were expressed as mean normalized expression (RLU)±SEM of three independent experiments performed in triplicates.

**Quantitative real time polymerase chain reactions**

RNA was isolated using TRIzol reagent (Life Technologies) according to manufacturers’ protocol. Quantitative real time polymerase chain reactions (QPCR) to detect mature miR-124 (Cat# 4427975, Assay ID 001182, Life Technologies) and miR-137 (Cat# 4427975, Assay ID 006016, Life Technologies) were performed using a TaqMan® MicroRNA Reverse Transcription Kit (Life Technologies) combined with TaqMan® 2x Universal PCR Master Mix (Life Technologies) and were normalized against RNU6B (Cat# 4427975, Assay ID 001093, Life Technologies). QPCR to detect mRNA was performed as described before ^28^ using the following primer sequences: α-tubulin forward: CCCTCGCCTTCTAACGCGTTGC, reverse: TGGTCTTGTCACTTGGCATCTGGC; nestin forward: GGGCAGCAACTGGCACACCTC, reverse: TGCAGCTTCAGCTTGGGGTCAG; SOX2 forward: GGAGACGGAGCTGAAGCCGC, reverse: CCGGGACCATACCATGAAGGCG; and DCX forward: TGCCTCAGGGAGTGCGCTACA, reverse: ACCAGTTGGGGTTGACATTCTTGGT; BCL2L13 forward: TCCTCTACGACTGCGTCTCT, reverse: TTGAACTCCTGGGGGTGAGG; BAX forward: GCGTGGTTGCCCTCTTCTAC, reverse: CCAGCCACCCTGGTCTTG.

**Immunohistochemistry and (confocal) microscopy**

The following antibodies were used: polyclonal chicken anti-GFP (Abcam, 1:500), monoclonal mouse anti-PSA-NCAM (Chemicon, 1:500) and polyclonal rabbit anti-BCL2L13 (Abcam, 1:200) or rabbit anti-cl-casp3 (Cell Signalling, 1:600) in combination with goat anti-chicken Alexa488 (Invitrogen, 1:500), goat anti-mouse Alexa647 (Invitrogen, 1:500) and goat anti-rabbit Alexa568 (Invitrogen, 1:500) respectively. Sections were counterstained for DNA using Hoechst (Invitrogen, 1:20000) to detect nuclei.

The SOX2 staining required an antigen retrieval treatment, performed by heating the sections in 0.1 M citrate buffer (pH 6.0) in a standard microwave (Samsung M6235) to a temperature of approximately 95°C for 15 minutes (5 minutes at 800 Watt, 5 minutes at 400 Watt and 5 minutes at 200 Watt). Antibodies used were polyclonal rabbit anti-SOX2 (Abgent, 1:500) and polyclonal goat anti-doublecortin C18 (DCX, Santa Cruz, 1:800) which were amplified with biotinylated goat anti-rabbit (Vector, 1:200) and biotinylated donkey anti-goat (1:500; Jackson ImmunoResearch Laboratories) respectively and avidin-biotin enzyme complex (ABC kit; Elite Vectastain, Brunschwig Chemie, Amsterdam, 1:1000) in combination with tyramide (1:500; 0.01% H2O2) and developed with diaminobenzidine (DAB; 20 mg/100 ml tris buffer; 0.01% H2O2). Images were acquired using a Leica CTR5500 microscope with the Leica MM AF program (MetaMorph, version 1.6.0), and stack images were acquired using the multidimensional acquisition. Brain sections from KA or SAL groups (n=3 each) were stained to detect the immature neuron marker DCX and the astrocyte marker GFAP and DCX^+^ cells were automatically identified and counted per section in z-projected confocal images using Cell Profiler ^34^. Immunoreactive cells were counted as described before using every tenth hippocampal section (of 40µm from 8 series) starting at the hippocampus, within each collected set of approximately 11 slices, as described before ^35^. The SGZ was defined as a two-cell body band around the limit of the GCL and the hilus, as described before ^36^. The granular zone (GZ) was defined as the area containing GCL+SGZ. Bar graphs represent normalized mean±SEM (% of BCL2L13^+^/Nestin-GFP^+^/PSA-NCAM^+^ of ctrl miR) or mean intensity±SD (background subtracted average gray value of BCL2L13 or cl-casp3 in Nestin-GFP^+^/PSA-NCAM^+^).

**Casp-3 activity**

Hippocampal NSPC were treated with varying concentrations of KA (Cat# K0250, Sigma-Aldrich). Staurosporine (Cat# A4400, Sigma-Aldrich) was used as a positive control for casp-3 activity. Lysates were collected according to manufacturer’s protocol and measured using a FLUOstar Optima plate reader (BMG Labtech GmbH).

**References**

1. Albrethsen, J. *et al.* Subnuclear proteomics in colorectal cancer: identification of proteins enriched in the nuclear matrix fraction and regulation in adenoma to carcinoma progression. *Mol Cell Proteomics* **9,** 988–1005 (2010).

2. Piersma, S. R. *et al.* Workflow comparison for label-free, quantitative secretome proteomics for cancer biomarker discovery: method evaluation, differential analysis, and verification in serum. *J. Proteome Res.* **9,** 1913–1922 (2010).

3. Pham, T. V., Piersma, S. R., Warmoes, M. & Jimenez, C. R. On the beta-binomial model for analysis of spectral count data in label-free tandem mass spectrometry-based proteomics. *Bioinformatics* **26,** 363–369 (2010).

4. Warmoes, M. *et al.* Proteomics of Mouse BRCA1-deficient Mammary Tumors Identifies DNA Repair Proteins with Potential Diagnostic and Prognostic Value in Human Breast Cancer. *Mol Cell Proteomics* **11,** M111.013334–M111.013334 (2012).

5. Gentleman, R. C. *et al.* Bioconductor: open software development for computational biology and bioinformatics. *Genome Biol* **5,** R80 (2004).

6. Verissimo, C. S. *et al.* Silencing of the microtubule-associated proteins doublecortin-like and doublecortin-like kinase-long induces apoptosis in neuroblastoma cells. *Endocr Relat Cancer* **17,** 399–414 (2010).

7. Liu, D.-Z. *et al.* Brain and blood microRNA expression profiling of ischemic stroke, intracerebral hemorrhage, and kainate seizures. *J Cereb Blood Flow Metab* **30,** 92–101 (2010).

8. Sempere, L. F. *et al.* Expression profiling of mammalian microRNAs uncovers a subset of brain-expressed microRNAs with possible roles in murine and human neuronal differentiation. *Genome Biol* **5,** R13 (2004).

9. Xiao, F. *et al.* miRecords: an integrated resource for microRNA-target interactions. *Nucleic Acids Res* **37,** D105–10 (2009).

10. Kiriakidou, M. *et al.* A combined computational-experimental approach predicts human microRNA targets. *Genes Dev* **18,** 1165–1178 (2004).

11. Rusinov, V., Baev, V., Minkov, I. N. & Tabler, M. MicroInspector: a web tool for detection of miRNA binding sites in an RNA sequence. *Nucleic Acids Res* **33,** W696–700 (2005).

12. Betel, D., Koppal, A., Agius, P., Sander, C. & Leslie, C. Comprehensive modeling of microRNA targets predicts functional non-conserved and non-canonical sites. *Genome Biol* **11,** R90 (2010).

13. Wang, X. & Naqa, El, I. M. Prediction of both conserved and nonconserved microRNA targets in animals. *Bioinformatics* **24,** 325–332 (2008).

14. Kim, S.-K., Nam, J.-W., Rhee, J.-K., Lee, W.-J. & Zhang, B.-T. miTarget: microRNA target gene prediction using a support vector machine. *BMC Bioinformatics* **7,** 411 (2006).

15. Yousef, M., Jung, S., Kossenkov, A. V., Showe, L. C. & Showe, M. K. Naïve Bayes for microRNA target predictions--machine learning for microRNA targets. *Bioinformatics* **23,** 2987–2992 (2007).

16. Krek, A. *et al.* Combinatorial microRNA target predictions. *Nat Genet* **37,** 495–500 (2005).

17. Kertesz, M., Iovino, N., Unnerstall, U., Gaul, U. & Segal, E. The role of site accessibility in microRNA target recognition. *Nat Genet* **39,** 1278–1284 (2007).

18. Miranda, K. C. *et al.* A pattern-based method for the identification of MicroRNA binding sites and their corresponding heteroduplexes. *Cell* **126,** 1203–1217 (2006).

19. Rehmsmeier, M., Steffen, P., Hochsmann, M. & Giegerich, R. Fast and effective prediction of microRNA/target duplexes. *RNA* **10,** 1507–1517 (2004).

20. Lewis, B. P., Burge, C. B. & Bartel, D. P. Conserved seed pairing, often flanked by adenosines, indicates that thousands of human genes are microRNA targets. *Cell* **120,** 15–20 (2005).

21. Yue, D., Liu, H. & Huang, Y. Survey of Computational Algorithms for MicroRNA Target Prediction. *Curr. Genomics* **10,** 478–492 (2009).

22. Sethupathy, P., Megraw, M. & Hatzigeorgiou, A. G. A guide through present computational approaches for the identification of mammalian microRNA targets. *Nat Meth* **3,** 881–886 (2006).

23. Peterson, S. M. *et al.* Common features of microRNA target prediction tools. *Front. Gene.* **5,** 23 (2014).

24. Brodersen, P. & Voinnet, O. Revisiting the principles of microRNA target recognition and mode of action. *Nat Rev Mol Cell Biol* **10,** 141–148 (2009).

25. Carmona-Saez, P., Chagoyen, M., Tirado, F., Carazo, J. M. & Pascual-Montano, A. GENECODIS: a web-based tool for finding significant concurrent annotations in gene lists. *Genome Biol* **8,** R3 (2007).

26. Nogales-Cadenas, R. *et al.* GeneCodis: interpreting gene lists through enrichment analysis and integration of diverse biological information. *Nucleic Acids Res* **37,** W317–22 (2009).

27. Tabas-Madrid, D., Nogales-Cadenas, R. & Pascual-Montano, A. GeneCodis3: a non-redundant and modular enrichment analysis tool for functional genomics. *Nucleic Acids Res* **40,** W478–83 (2012).

28. Fitzsimons, C. P. *et al.* Knockdown of the glucocorticoid receptor alters functional integration of newborn neurons in the adult hippocampus and impairs fear-motivated behavior. *Mol Psychiatry* **18,** 993–1005 (2012).

29. Hebert, C., Norris, K., Scheper, M. A., Nikitakis, N. & Sauk, J. J. High mobility group A2 is a target for miRNA-98 in head and neck squamous cell carcinoma. *Mol Cancer* **6,** 5 (2007).

30. Ng, Y. S., Roca, H., Fuller, D., Sud, S. & Pienta, K. J. Chemical transfection of dye-conjugated microRNA precursors for microRNA functional analysis of M2 macrophages. *J. Cell. Biochem.* **113,** 1714–1723 (2012).

31. Tay, Y., Zhang, J., Thomson, A. M., Lim, B. & Rigoutsos, I. MicroRNAs to Nanog, Oct4 and Sox2 coding regions modulate embryonic stem cell differentiation. *Nature* **455,** 1124–1128 (2008).

32. Kataoka, T. *et al.* Bcl-rambo, a novel Bcl-2 homologue that induces apoptosis via its unique C-terminal extension. **276,** 19548–19554 (2001).

33. Sah, D. W., Ray, J. & Gage, F. H. Regulation of voltage- and ligand-gated currents in rat hippocampal progenitor cells in vitro. *J Neurobiol* **32,** 95–110 (1997).

34. Carpenter, A. E. *et al.* CellProfiler: image analysis software for identifying and quantifying cell phenotypes. *Genome Biol* **7,** R100 (2006).

35. van Hooijdonk, L. W. A. *et al.* Lentivirus-mediated transgene delivery to the hippocampus reveals sub-field specific differences in expression. *BMC Neurosci* **10,** 2 (2009).

36. Espósito, M. S. *et al.* Neuronal differentiation in the adult hippocampus recapitulates embryonic development. *J Neurosci* **25,** 10074–10086 (2005).

**Legends to supplementary figures and tables**

**Supplementary Figure 1 – Hallmarks of KA-SE in the mouse DG and proteomics quality control.**

(**A-D**) Representative confocal images showing the temporal increase in DCX^+^ immunoreactive cells observed in the DG 0, 1, 3 and 7 days after KA-SE. Right images show higher magnifications of the images depicted left.

(**E**) Bar graph showing the quantification of DCX^+^ cells three days after KA-SE. Values represent normalized mean (% of control) ±SEM (n=3) and were tested using unpaired Student’s *t*-test (* *p* < 0.05).

(**F**) Representative confocal images showing the temporal increase in astrocytic marker GFAP immunoreactivity observed in the DG shortly after KA-SE.

Control: 0 days after KA-SE. Scale bars in (**A-D**) and (**F**): 50µm.

(**G**) SDS PAGE gel image showing separation pattern of KA and SAL proteins.

(**H**) Venn diagrams indicate the overlap between replicates (n=3) for the SAL and KA groups and between the two groups.

(**I)** For the proteins identified in 3/3 samples the average coefficient of variance (CV) for protein quantification was calculated to be 21.5% for the SAL group and 24.6% for the KA group, indicating good quantitative reproducibility.

**Supplementary Figure 2 – Gene networks identified around the significantly dysregulated proteins in the DG shortly after KA-SE.**

(**A**) Global protein network of upregulated proteins in KA-SE samples.

(**B**) and (**C)** Two of the most significantly overrepresented gene networks depicting nodes around (non-regulated; white) NFkB and TNF with direct (solid edge) and indirect (dashed edge) interactions of significantly (red) upregulated proteins.

(**D**) Global protein network of downregulated proteins in KA-SE samples.

(**E**) Significantly overrepresented gene network depicting nodes around NFkB and significantly downregulated proteins with direct or indirect interactions.

(**F)** Significantly overrepresented gene network depicting nodes around TP53, including NDUFB6, NDUFB7 and significantly downregulated proteins with direct or indirect interactions.

Gene products (nodes) are represented as standard IPA polygons and relationships with lines (edges) between nodes. Intensity of the node color indicates the degree of regulation (SAL vs. KA) and relationship strength is inversely related to line length. Genes in uncolored nodes were not identified as differentially expressed in our experiments and were integrated by the IPA knowledge database. Full lines indicate a direct interaction and dashed lines an indirect interaction. Arrows represent activation while non-arrowed lines binding only.

**Supplementary Figure 3 – Validation of relevant changes detected in proteomics, transcriptomics and miR profiling experiments.**

(**A**) Validation of changes in BCL2L13 protein levels by WB. Representative immunoblots and quantifications show that KA-SE induced a significant reduction in BCL-RAMBO protein levels in the DG (* *p* < 0.05).

(**B**) Validation of changes in BAX protein levels by WB. Representative immunoblots and quantifications show that KA-SE induced a significant reduction in BAX protein levels in the DG (* *p* < 0.05).

(**C**) Pearson correlation analysis of all the proteins significantly upregulated by KA-SE and their corresponding mRNAs showing no significant correlation (Pearson r = -0.2147, *p* > 0.05).

(**D**) Pearson correlation analysis of all the proteins significantly downregulated by KA-SE and their corresponding mRNAs showing no significant correlation (Pearson r = -0.1647, *p* > 0.05).

(**E**) Validation of changes in BCL2L13 mRNA levels by Q-PCR, showing that KA-SE didn’t induce significant changes in levels of BCL2L13 mRNA in the DG after 3 days (*p* > 0.05).

(**F**) Validation of changes in BAX mRNA levels by Q-PCR, showing that KA-SE induced significant changes in levels of BAX mRNA in the DG after 3 days (**p* < 0.05).

(**G**) Validation of changes in miR-124 levels by TaqMan Q-PCR, showing that KA-SE induced significant changes in levels of miR-124 in the DG after 3 days (****p* < 0.001).

(**H**) Validation of changes in miR-137 levels by TaqMan Q-PCR, showing that KA-SE induced significant changes in levels of miR-137 in the DG after 3 days (****p* < 0.001).

Values in bar graphs represent normalized mean expression or fold change±SEM (N=3) and were tested using unpaired Student’s *t*-test. Pearson correlation analysis was performed using GraphPad Prism 5.0.

**Supplementary Figure 4 – Biological pathways overrepresented among significantly dysregulated proteins in the DG.**

(**A**) GO analysis of the proteins upregulated in the DG after KA-SE.

(**B**) GO analysis of the proteins downregulated in the DG after KA-SE.

BPs were identified with Genocodis modular enrichment GO analysis. Red bars: number of annotated members per BP. Blue bars: hypergeometric FDR corrected *p*-values. Two or more regulated proteins per process were used as inclusion criterion.

(**C**) IPA showing the downregulated proteins Akt3, BAX and BCL2L13 within a mitochondrial pathway of apoptosis. Gene products, relationships, degree of regulation, relationship strength and interactions are represented as described in Fig. 2

**Supplementary Figure 5 - Genecodis GO analysis of mmu-miR-9, mmu-miR-125a-3p, mmu-miR-125b-3p, mmu-miR-135a, mmu-miR-135b and mmu-miR-190 that share common predicted targets with mmu-miR-124.**

(**A**) Venn diagram showing miRecords database predictions of miR-mRNA interaction mining for miR-124 (2119 Genecodis GO annotated targets) and miR-9 (2823 Genecodis GO annotated targets) with ≥3 algorithms positively predicting an miR-mRNA binding. The miR-124 and miR-9 pair had 690 predicted common Genecodis GO annotated targets. The bar graph depicts Genecodis Comparative Modular Gene Enrichment GO analysis of the 690 predicted common targets between miR-124 and miR-9. Apoptosis is among the significantly (Hypergeometric FDR corrected *p* = 1.26*10^-4^) overrepresented GO biological processes with BCL2L13 as a member.

(**B**) Schematic representation of the BCL2L13 3’UTR with miR-124 and miR-9 binding regions highlighted, none of which meet the required inclusion criteria for cooperative action with miR-124. Details of cooperativity predictions are given in Table S9.

(**C**) Venn diagram showing miRecords database predictions of miR-mRNA interaction mining for miR-124 (2119 Genecodis GO annotated targets) and miR-125a-3p (2380 Genecodis GO annotated targets) with ≥3 algorithms positively predicting an miR-mRNA binding. The miR-124 and miR-125a-3p pair had 585 predicted common Genecodis GO annotated targets. The bar graph depicts Genecodis Comparative Modular Gene Enrichment GO analysis of the 585 predicted common targets between miR-124 and miR-125a-3p. Apoptosis is among the significantly (Hypergeometric FDR corrected *p* = 1.55*10^-3^) overrepresented GO biological processes with BCL2L13 as a member.

(**D**) Schematic representation of the BCL2L13 3’UTR with miR-124 and miR-125a-3p binding regions highlighted, none of which meet the required inclusion criteria for cooperative action with miR-124. Details of cooperativity predictions are given in Table S9.

(**E**) Venn diagram showing miRecords database predictions of miR-mRNA interaction mining for miR-124 (2119 Genecodis GO annotated targets) and miR-125b-3p (470 Genecodis GO annotated targets) with ≥3 algorithms positively predicting an miR-mRNA binding. The miR-124 and miR-125b-3p pair had 139 predicted common Genecodis GO annotated targets. The bar graph depicts Genecodis Comparative Modular Gene Enrichment GO analysis of the 139 predicted common targets between miR-124 and miR-125b-3p. Apoptosis is not among the significantly (Hypergeometric FDR corrected *p* > 0.05) overrepresented GO biological processes.

(**F**) Schematic representation of the BCL2L13 3’UTR with miR-124 and miR-125b-3p binding regions highlighted, none of which meet the required inclusion criteria for cooperative action with miR-124. Details of cooperativity predictions are given in Table S9.

(**G**) Venn diagram showing miRecords database predictions of miR-mRNA interaction mining for miR-124 (2119 Genecodis GO annotated targets) and miR-135a (2439 Genecodis GO annotated targets) with ≥3 algorithms positively predicting an miR-mRNA binding. The miR-124 and miR-135a pair had 633 predicted common Genecodis GO annotated targets. The bar graph depicts Genecodis Comparative Modular Gene Enrichment GO analysis of the 633 predicted common targets between miR-124 and miR-135a. Apoptosis is among the significantly (Hypergeometric FDR corrected *p* = 2.30*10^-3^) overrepresented GO biological processes with BCL2L13 as a member.

(**H**) Schematic representation of the BCL2L13 3’UTR with miR-124 and miR-135a binding regions highlighted. The predicted miR-124 binding region at position 916 and the predicted miR-135a binding region at position 848 met the required inclusion criteria for cooperative action. Details of cooperativity predictions are given in Table S9.

(**I**) Venn diagram showing miRecords database predictions of miR-mRNA interaction mining for miR-124 (2119 Genecodis GO annotated targets) and miR-135b (2473 Genecodis GO annotated targets) with ≥3 algorithms positively predicting an miR-mRNA binding. The miR-124 and miR-135b pair had 643 predicted common Genecodis GO annotated targets. The bar graph depicts Genecodis Comparative Modular Gene Enrichment GO analysis of the 643 predicted common targets between miR-124 and miR-135b. Apoptosis is among the significantly (Hypergeometric FDR corrected *p* = 3.88*10^-4^) overrepresented GO biological processes with BCL2L13 as a member.

(**J**) Schematic representation of the BCL2L13 3’UTR with miR-124 and miR-135b binding regions highlighted, none of which meet the required inclusion criteria for cooperative action with miR-124. Details of cooperativity predictions are given in Table S9.

(**K**) Venn diagram showing miRecords database predictions of miR-mRNA interaction mining for miR-124 (2119 Genecodis GO annotated targets) and miR-190 (1025 Genecodis GO annotated targets) with ≥3 algorithms positively predicting an miR-mRNA binding. The miR-124 and miR-190 pair had 277 predicted common Genecodis GO annotated targets. The bar graph depicts Genecodis Comparative Modular Gene Enrichment GO analysis of the 277 predicted common targets between miR-124 and miR-190. Apoptosis is among the significantly (Hypergeometric FDR corrected *p* = 1.12*10^-2^) overrepresented GO biological processes.

(**L**) Schematic representation of the BCL2L13 3’UTR with miR-124. The mouse BCL-RAMBO 3’UTR was not predicted to contain miR-190 binding regions, instead a putative binding region was found in the ORF. Details of cooperativity predictions are given in Table S9.

**Supplementary Figure 6 – Validation of exogenous expression of FLAG-hBCL2L13 in hippocampal NSPC cultures.**

(**A**) Representative immunoblots and bar graph showing quantifications of endogenous BCL2L13 (endogenous-BCL2L13) expression or FLAG-tagged hBCL2L13 (FLAG-hBCL2L13) after transfection. Transfection significantly increases FLAG immunoreactivity (** *p* <0.01) without affecting endogenous-BCL2L13 (ns, *p*  > 0.05).

(**B**) Representative immunoblots and bar graph showing quantifications of BCL2L13 expression after siSCR (scramble siRNA) and siBCL2L13 transfection and subsequent veh/KA treatment. siBCL2L13 transfection significantly decreases BCL2L13 immunoreactivity (** *p* <0.01) without being affected by veh/KA treatments (ns, *p*  > 0.05).

(**C**) Representative immunoblots and bar graph showing quantifications of pro- and cl-casp3 expression after siSCR (scramble siRNA) and siBCL2L13 transfection and subsequent KA treatment. Transfection with both 100nM siSCR and siBCL2L13 did not result in detectable levels cl-casp3 when treated with veh. When treated with 30µM KA, siSCR transfected NSPC showed a significant increase in cl-casp3 levels (* *p* < 0.05, compared to siSCR+veh; ** *p* < 0.05, compared to siSCR+KA). When treated with 30µM KA, siBCL2L13 transfected NSPC showed a significant abolishement of this KA induced increase in cl-casp3 levels (* *p* < 0.05, compared to siSCR+veh; ** *p* < 0.05, compared to siSCR+KA).

**Supplementary Figure 7 - Full-length blots corresponding to Figure 6 and 8B.**

Representative full-lenght immunoblots showing the effect of miR-124 and miR-137 alone or in combination on endogenous BCL2L13 protein levels (A, corresponding to data presented in Figure 6) and on pro- and cleaved-caspase3 levels (B, corresponding to data presented in Figure 8B) in hippocampal NSPC cultures. Lanes marked with a dash (-) contained samples irrelevant for the experiments described in the figures. All samples were run on the same gel and transferred to the same membrane, which was cut after transfer at the position indicated by the horizontal black line to avoid possible antibody cross-reactivity during immunoblotting. Molecular weights (kD) were estimated using Precision Plus Dual Color Standards (Bio-Rad), indicated as MWM.

**Supplementary Table 1 - List of peptide identifications measured by proteomics after KA-SE in the DG.**

**Supplementary Table 2 - Significantly dysregulated proteins measured by proteomics after KA-SE in the DG.**

**Supplementary Table 3 - Genecodis GO analysis of KA-SE induced significantly upregulated proteins.**

**Supplementary Table 4 - Genecodis GO analysis of KA-SE induced significantly downregulated proteins.**

**Supplementary Table 5 – Transcriptomic changes after KA-SE in the DG.**

**Supplementary Table 6 – miR profiling after KA-SE in the DG.**

**Supplementary Table 7 - List of miRECORDs predicted mmu-miR-124 targets.**

**Supplementary Table 8 - List of miRECORDs predicted mmu-miR-137 targets.**

**Supplementary Table 9 - miRECORDs and RNA22 lists of predicted miR binding regions in BCL2L13’s 3’UTR.** Inclusion criteria for predicted cooperative action together with miR-124 were: miR among the upregulated brain enriched or specific miRs (Figure 4C), miR with at least a 7mer binding base pairing in the UTR, miR binding region within 80nt proximity of miR-124’s first or last bp binding regions (between 1520-1700 or between 2318-2498).

**Supplementary Table 10 - Genecodis GO analysis of the 336 mmu-miR-124 and mmu-miR-137 miRECORDs predicted common targets.** Apoptosis is among the significantly (*p* = 1.65*10^-10^) overrepresented GO biological processes with BCL2L13 as a member.

**Supplementary Table 11 - List of miRECORDs predicted mmu-miR-9 target mRNAs and Genecodis GO analysis of the 690 mmu-miR-124 and mmu-miR-9 predicted common targets.** Apoptosis is among the significantly (*p* = 1.26*10^-4^) overrepresented GO biological processes with BCL2L13 as a member.

**Supplementary Table 12 - List of miRECORDs predicted mmu-miR-125a-3p target mRNAs and Genecodis GO analysis of the 585 mmu-miR-124 and mmu-miR-125a-3p common targets.** Apoptosis is among the significantly (*p* = 1.55*10^-3^) overrepresented GO biological processes with BCL2L13 as a member.

**Supplementary Table 13 - List of miRECORDs predicted mmu-miR-125b-3p target mRNAs and Genecodis GO analysis of the 139 mmu-miR-124 and mmu-miR-125b-3p common targets.** Apoptosis is not among the significantly overrepresented GO biological processes (*p* > 0.05). Bioinformatics analysis predicted binding regions for mmu-miR-125b-3p were predicted in BCL2L13 3’UTR.

**Supplementary Table 14 - List of miRECORDs predicted mmu-miR-135a target mRNAs and Genecodis GO analysis of the 633 mmu-miR-124 and mmu-miR-135a common targets.** Apoptosis is among the significantly (*p* = 2.30*10^-3^) overrepresented GO biological processes with BCL2L13 as a member.

**Supplementary Table 15 - List of miRECORDs predicted mmu-miR-135b target mRNAs. Genecodis GO analysis of the 643 mmu-miR-124 and mmu-miR-135b common targets.** Apoptosis is among the significantly (*p* = 3.88*10^-4^) overrepresented GO biological processes with BCL2L13 as a member.

**Supplementary Table 16 - List of miRECORDs predicted mmu-miR-190 target mRNAs and Genecodis GO analysis of the 277 mmu-miR-124 and mmu-miR-190 common targets.** Apoptosis is among the significantly (*p* = 1.12*10^-2^) overrepresented GO biological processes with BCL2L13 as a member.
