# Supplementary figures and images for: MicroRNA-124 and -137 cooperativity controls caspase-3 activity through BCL2L13 in hippocampal neural stem cells
Source: Sci Rep. 2015 Jul 24;5:12448. doi: 10.1038/srep12448 (PMC4513647; doi:10.1038/srep12448)

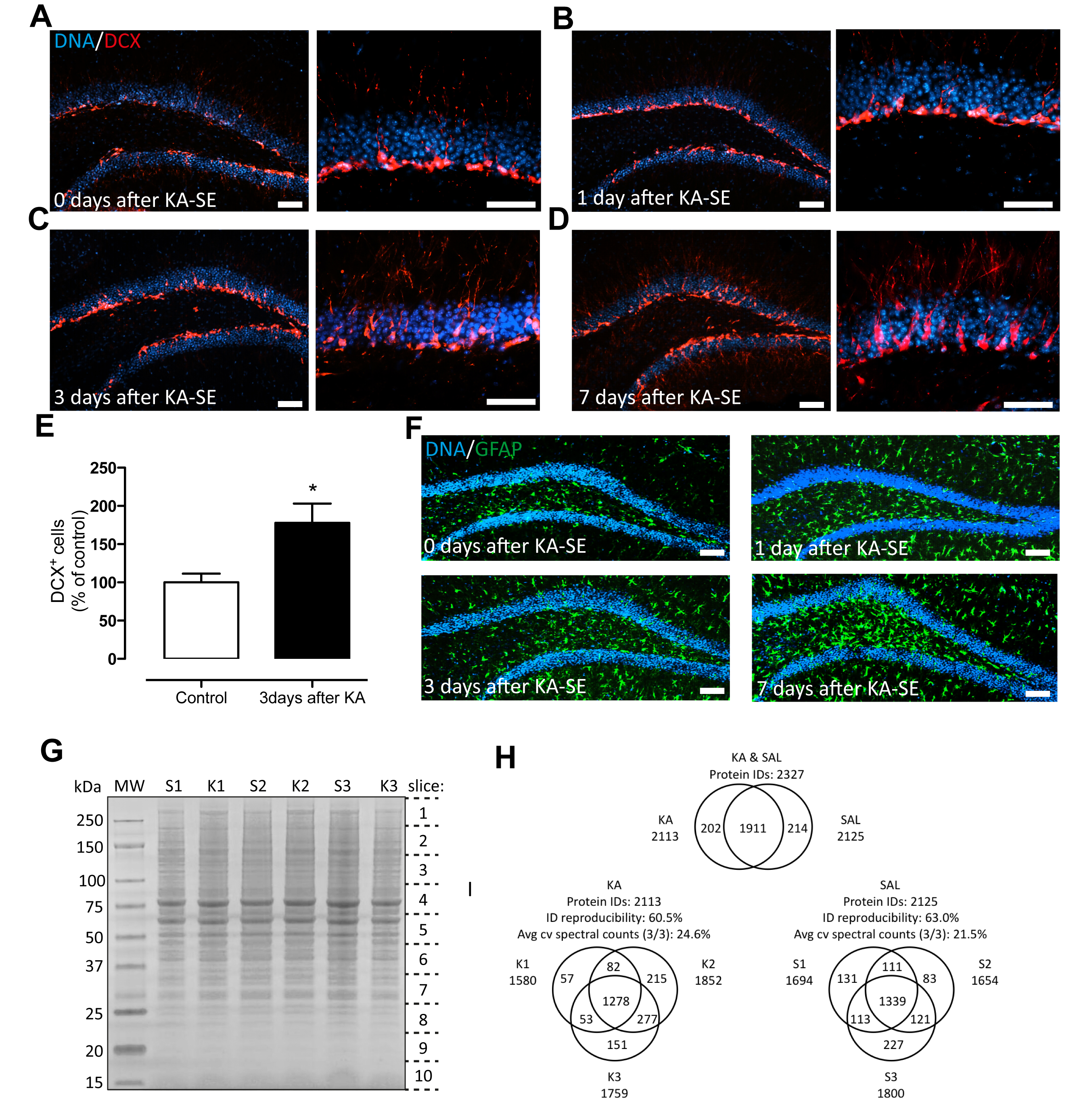

Supplement: Supplementary Figure 1 [file srep12448-s2.tiff]

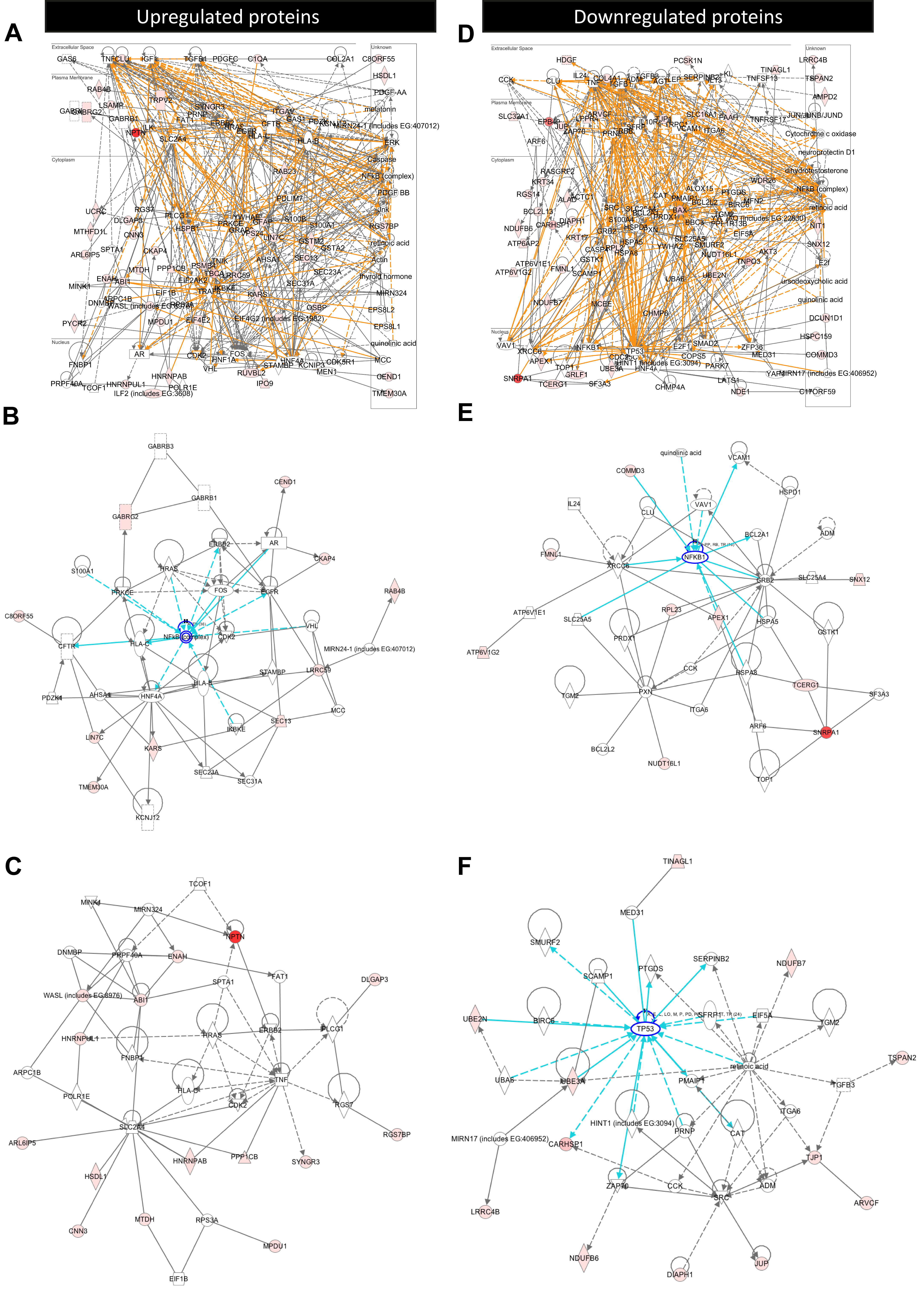

Supplement: Supplementary Figure 2 [file srep12448-s3.tiff]

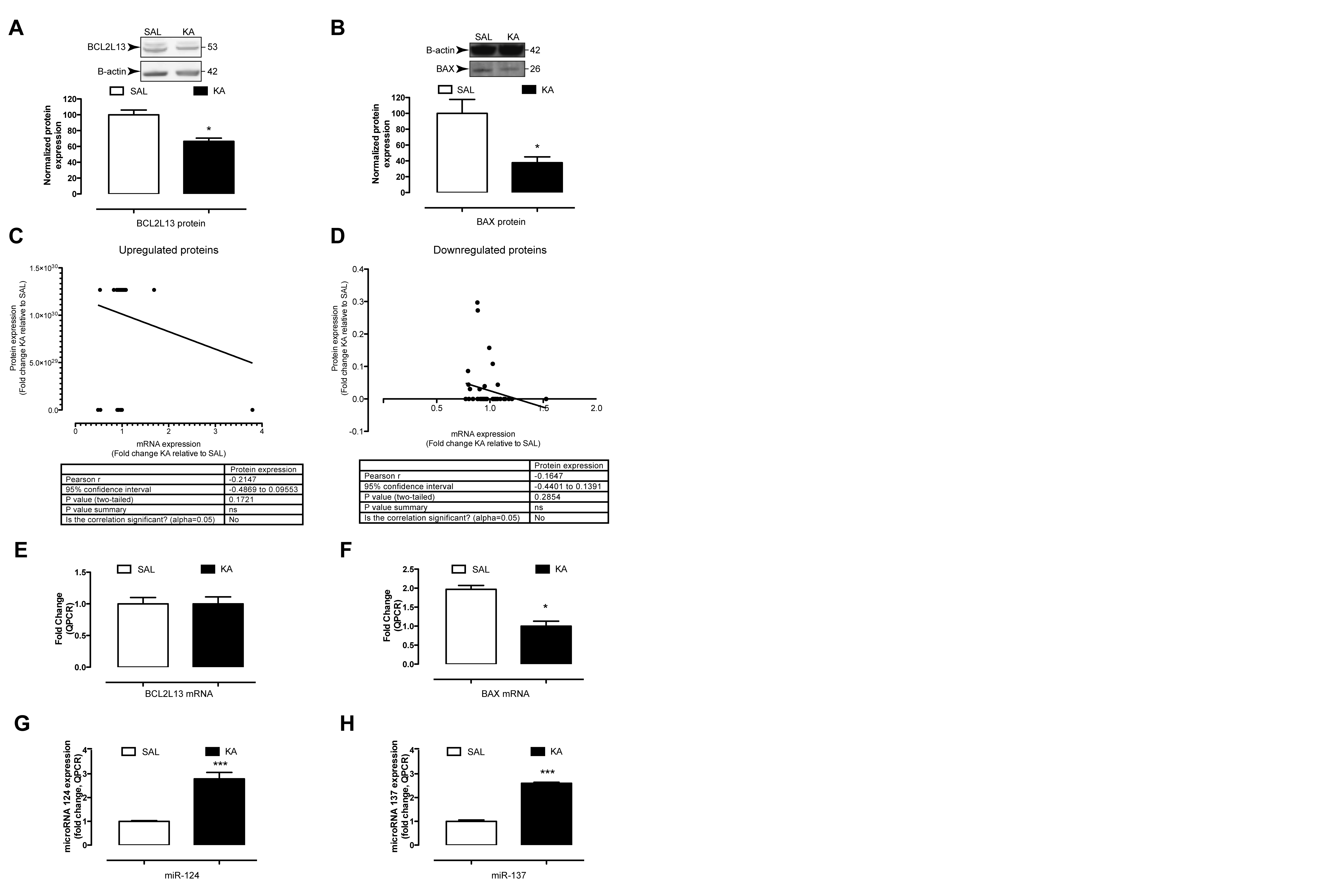

Supplement: Supplementary Figure 3 [file srep12448-s4.tiff]

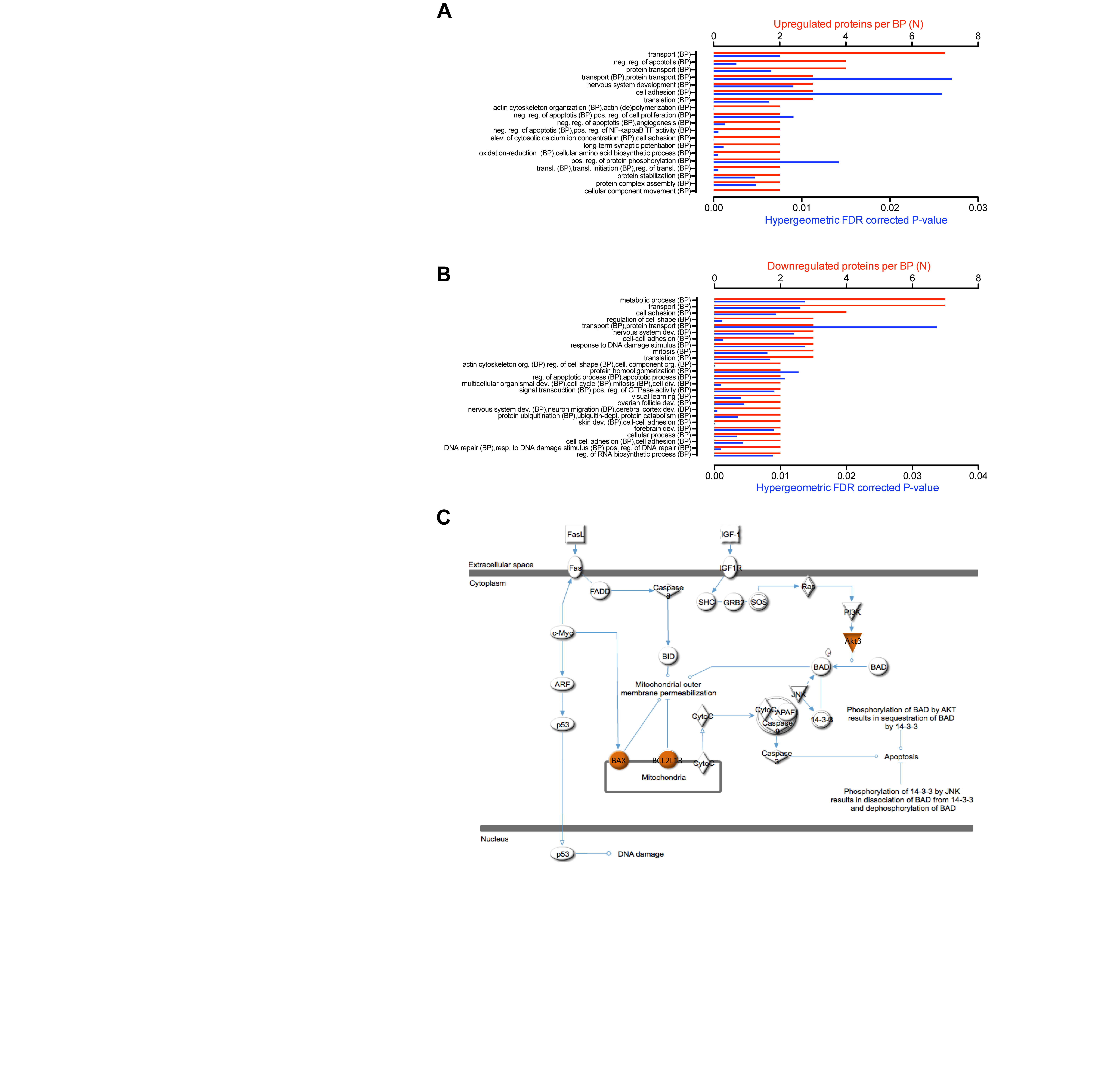

Supplement: Supplementary Figure 4 [file srep12448-s5.tiff]

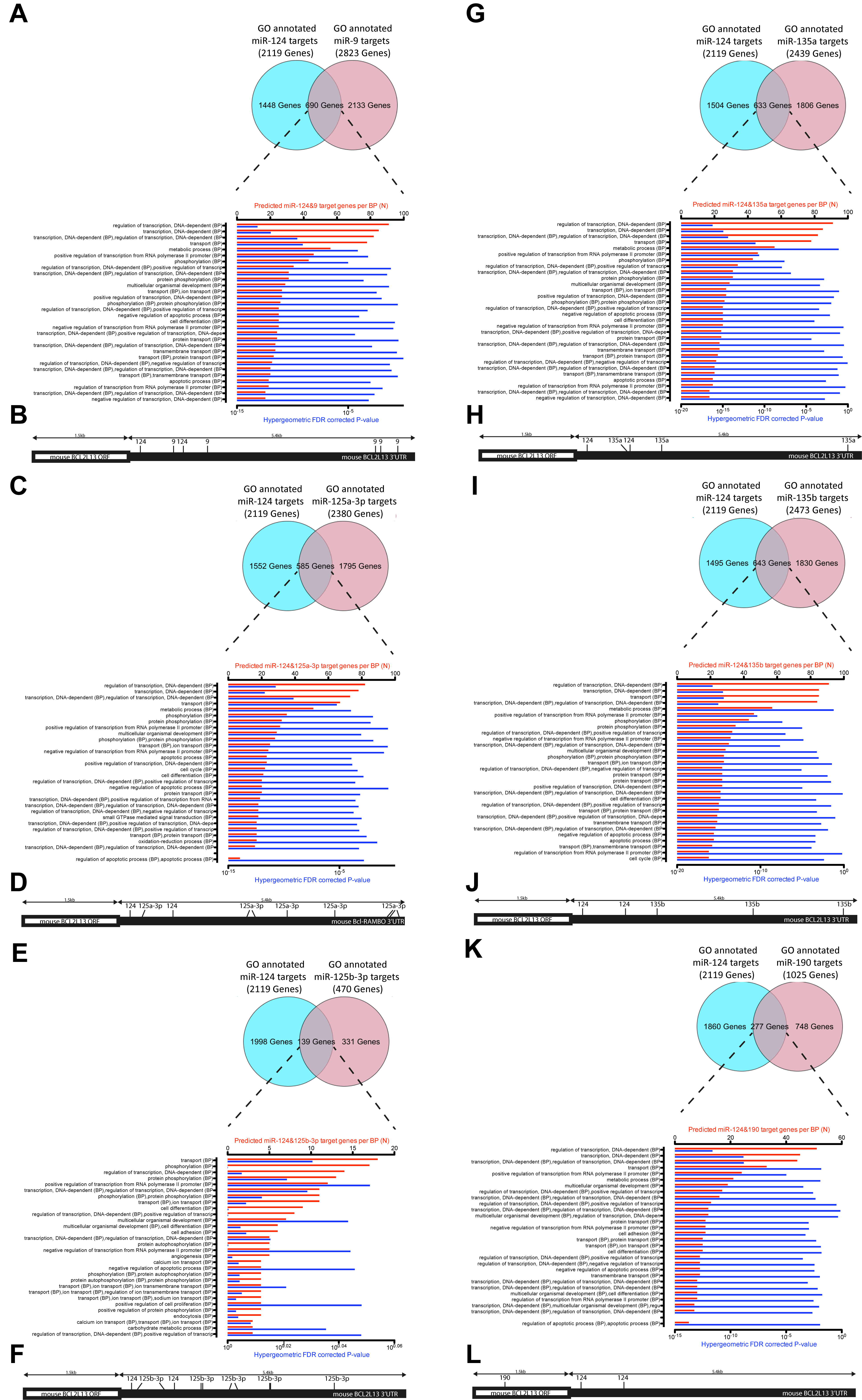

Supplement: Supplementary Figure 5 [file srep12448-s6.tiff]

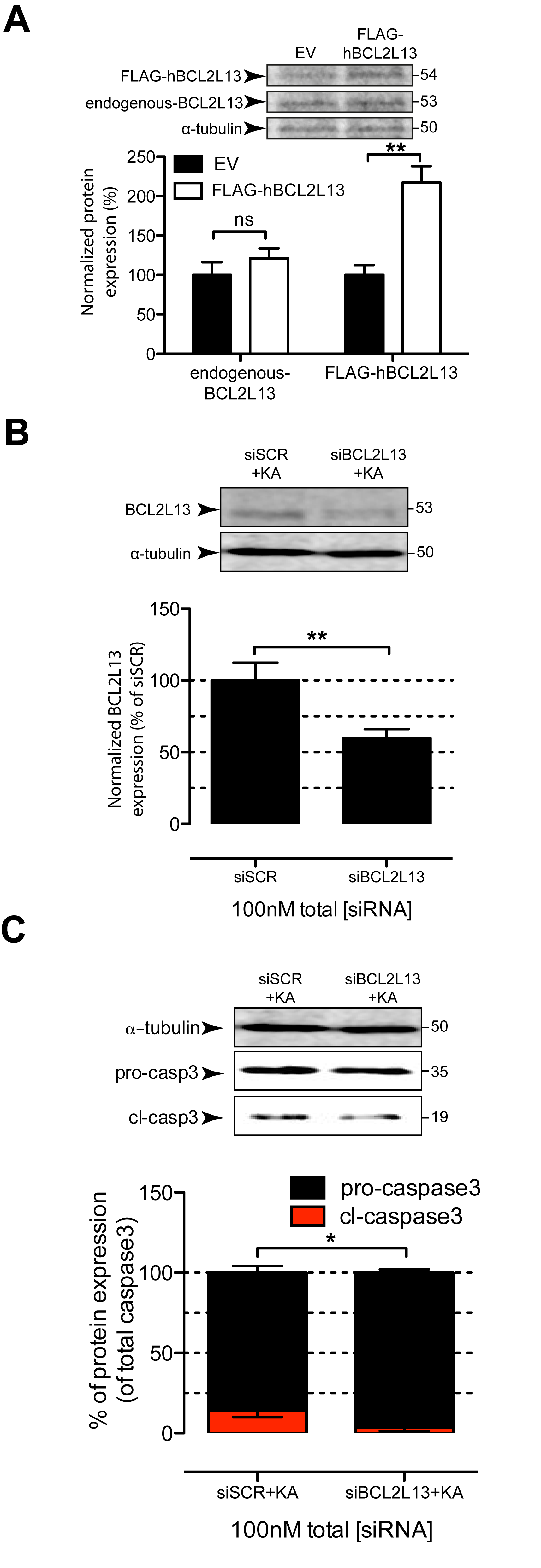

Supplement: Supplementary Figure 6 [file srep12448-s7.tiff]

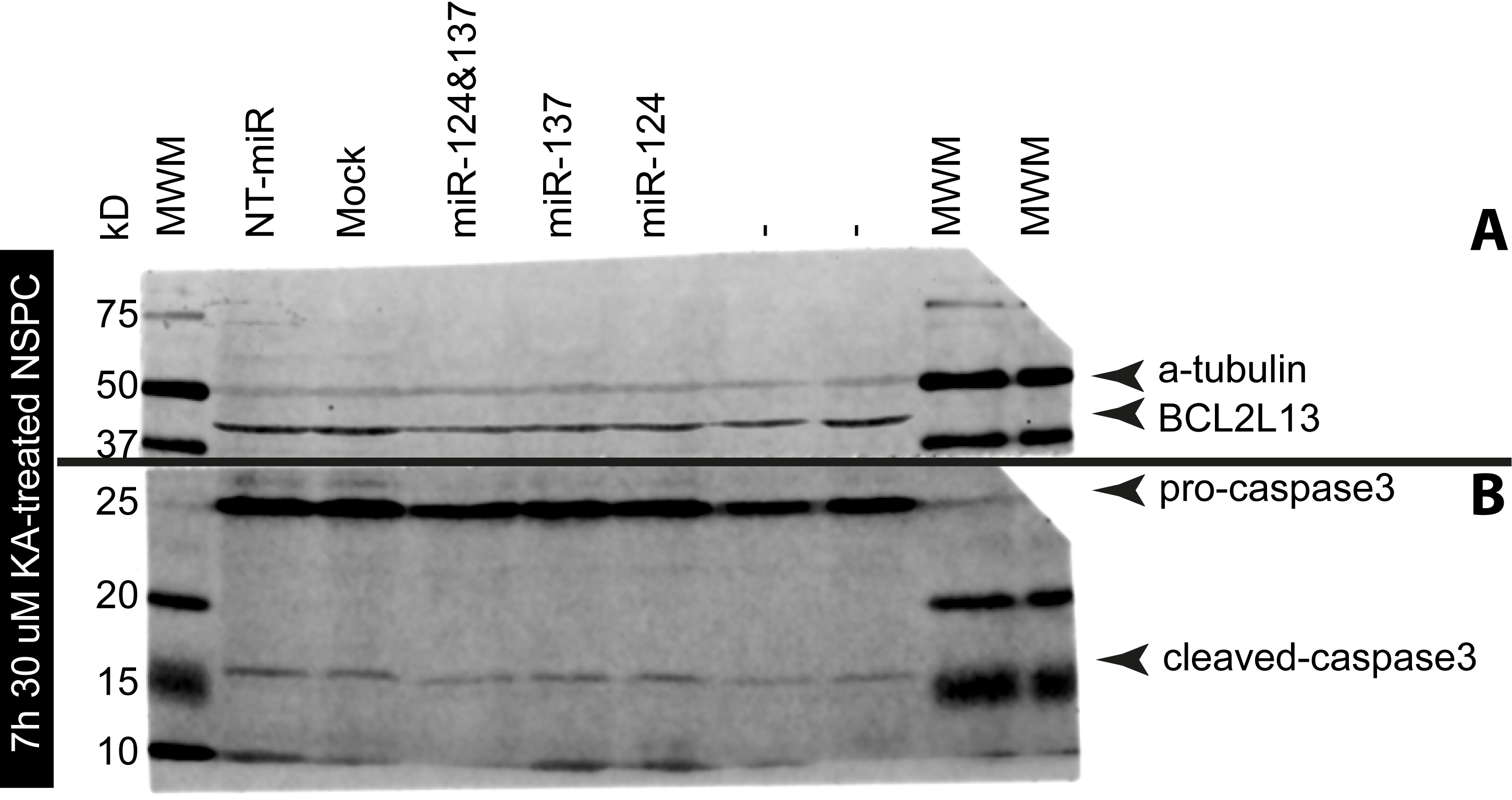

Supplement: Supplementary Figure 7 [file srep12448-s8.tiff]
